# The case and context for atmospheric methane as an exoplanet biosignature

**DOI:** 10.1073/pnas.2117933119

**Published:** 2022-03-30

**Authors:** Maggie A. Thompson, Joshua Krissansen-Totton, Nicholas Wogan, Myriam Telus, Jonathan J. Fortney

**Affiliations:** ^a^Department of Astronomy and Astrophysics, University of California, Santa Cruz, CA 95064;; ^b^Department of Earth and Space Sciences, University of Washington, Seattle, WA 98195;; ^c^Department of Earth and Planetary Sciences, University of California, Santa Cruz, CA 95064

**Keywords:** methane, biosignatures, planetary atmospheres

## Abstract

Methane has been proposed as an exoplanet biosignature. Imminent observations with the James Webb Space Telescope may enable methane detections on potentially habitable exoplanets, so it is essential to assess in what planetary contexts methane is a compelling biosignature. Methane’s short photochemical lifetime in terrestrial planet atmospheres implies that abundant methane requires large replenishment fluxes. While methane can be produced by a variety of abiotic mechanisms such as outgassing, serpentinizing reactions, and impacts, we argue that—in contrast to an Earth-like biosphere—known abiotic processes cannot easily generate atmospheres rich in CH_4_ and CO_2_ with limited CO due to the strong redox disequilibrium between CH_4_ and CO_2_. Methane is thus more likely to be biogenic for planets with 1) a terrestrial bulk density, high mean-molecular-weight and anoxic atmosphere, and an old host star; 2) an abundance of CH_4_ that implies surface fluxes exceeding what could be supplied by abiotic processes; and 3) atmospheric CO_2_ with comparatively little CO.

## Significance

Astronomers will soon begin searching for biosignatures, atmospheric gases or surface features produced by life, on potentially habitable planets. Since methane is the only biosignature that the James Webb Space Telescope could readily detect in terrestrial atmospheres, it is imperative to understand methane biosignatures to contextualize these upcoming observations. We explore the necessary planetary context for methane to be a persuasive biosignature and assess whether, and in what planetary environments, abiotic sources of methane could result in false-positive scenarios. With these results, we provide a tentative framework for assessing methane biosignatures. If life is abundant in the universe, then with the correct planetary context, atmospheric methane may be the first detectable indication of life beyond Earth.

The next phase of exoplanet science will focus on characterizing exoplanet atmospheres, including those of potentially habitable planets. For example, the James Webb Space Telescope (JWST) will be capable of characterizing the atmospheres of transiting, terrestrial planets around low-mass stars, such as the TRAPPIST-1 system (1, 2). A new class of ground-based telescopes (3) may be able to detect atmospheric constituents such as oxygen, water, and carbon dioxide on nearby rocky exoplanets via high-resolution spectroscopy (4). In subsequent decades, the Astro2020 Decadal Survey report has prioritized a large infrared/optical/ultraviolet (UV) telescope built to search for signs of life—biosignatures—on ∼25 habitable-zone planets (5). Life may modify its planetary environment in multiple ways, including producing waste gases that alter a planet’s atmospheric composition. As a result, an understanding of detectable biogenic waste gases and their nonbiological false positives is needed.

Terrestrial planets, which are the focus of this study, require significant methane surface fluxes to sustain high atmospheric abundances. On Earth, life sustains large methane surface fluxes, and so methane has long been regarded as a potential biosignature gas for terrestrial exoplanets. Previous studies have considered abiotic methane production (6–11), methane biosignatures in the context of chemical disequilibrium (12–15), and prospects for remote detection of methane in terrestrial atmospheres (6, 9, 15–17). During the Archean eon (4 to 2.5 Ga), Earth’s atmosphere likely had high methane abundances (∼10^2^ to 10^4^ times modern) due to life (i.e., methanogenesis) (8, 18, 19). Methane is thus not a hypothetical biosignature because we know of an inhabited terrestrial planet with detectable levels of biogenic methane—the Archean Earth. However, methane is sometimes dismissed as irredeemably ambiguous due to its ubiquity in planetary environments and potential for nonbiological production (8, 9). Additional work is clearly needed to understand methane biosignatures and their false positives within different planetary contexts.

While other studies have reviewed the biosignature gases oxygen (20), phosphine (21), isoprene (22), and ammonia (23), in the near term, these gases will likely be difficult to detect or will be detectable only in extended H_2_-dominated atmospheres on planets with large biogenic fluxes. In contrast, for Earth-like biogenic fluxes, methane is one of the few biosignatures that may be readily detectable with JWST (24–26). For example, biological methane on an early Earth-like TRAPPIST-1e could be detectable with 5 to 10 transits with JWST (17, 27) and would remain detectable even with an optically thick aerosol layer at 10 to 100 mbar, assuming plausible instrument noise and negligible stellar contamination (17).

Given the imminent feasibility of observing methane with JWST, it is imperative to determine the planetary conditions where methane is a compelling biosignature. Despite the patchwork of past studies on methane biosignatures, a recent and dedicated investigation into the conditions needed for atmospheric methane to be a good exoplanet biosignature is lacking. This study provides an updated assessment of the necessary planetary context for methane biosignatures. First, we present the case for methane as a biosignature, including its short photochemical lifetime and relationship with chemical disequilibrium and CO antibiosignatures. We then explore the possibility of abiotic methane fluxes as large as those caused by known biogenic sources, in part using different modeling tools. We also discuss the purported presence of methane on Mars and simulate atmospheric methane on temperate Titan-like exoplanets. Based on these results, we propose a framework for identifying methane biosignatures and discuss detectability prospects with next-generation missions.

## Biological Methane Production on Earth

The vast majority of methane in Earth’s atmosphere today, and throughout most of its history, is biogenic. At present, Earth’s ∼30 Tmol/y global methane emissions are predominantly produced directly by life (including anthropogenic sources), and most of the rest is thermogenic methane that derives from previous life, such as metamorphic reactions of organic matter (28). Genuinely abiotic methane emissions, while uncertain, are comparatively tiny (28).

Biological methane production, or methanogenesis, is a simple metabolism performed by anaerobic microbes (i.e., those not requiring oxygen for growth). Methanogenic microbes can be divided into three groups: hydrogenotrophic (reaction **1**), acetoclastic (reaction **2**), and methylotrophic methanogens:

[1] $$CO_{2}+4H_{2}\to CH_{4}+2H_{2}O$$

[2] $$CH_{3}\mathrm{COOH}\to CH_{4}+CO_{2}.$$

Hydrogenotrophic methanogens typically oxidize H_2_ and reduce CO_2_ to CH_4_ and contribute approximately one-third of current biogenic methane emissions. Acetoclastic methanogens use acetate, contributing approximately two-thirds of current biogenic methane emissions; and finally, methylotrophic methanogens use methylated compounds but do not contribute significantly to global biogenic methane emissions (29). Methane can also be produced indirectly by life as a byproduct of degrading organic matter from dead organisms, called “thermogenic methane.”

If life elsewhere is common, methanogenesis may be widespread due to the likely ubiquity of the CO_2_ + H_2_ redox couple in terrestrial planet atmospheres and the potential metabolic payoff from exploiting such commonly outgassed substrates. Methanogenesis is an ancient metabolism on Earth with phylogenetic analyses implying that methanogenesis originated between 4.11 and 3.78 Ga and reconstructions of the last universal common ancestor suggesting methanogens were one of the earliest lifeforms to evolve on Earth (30–32).

There are several reasons to expect methane-cycling biospheres to produce large CH_4_ fluxes. During the Archean, xenon isotopes—which ostensibly reflect abundances of escaping, hydrogen-bearing species in the upper atmosphere—likely imply large methane abundances (>0.5%) (19, 33). This Xe isotope fractionation can potentially be explained by another hydrogen-bearing species (e.g., >1% H_2_ or >1% H_2_O), but such explanations are tentatively disfavored: Catling and Zahnle (19) and Kadoya and Catling (34) place an upper limit of H_2_ in the Archean atmosphere of 1% and other paleo-pressure and surface temperature estimates likely preclude >1% H_2_O above the tropopause. Moreover, multiple ecosystem models for the Archean Earth estimate large biogenic CH_4_ fluxes and abundant atmospheric CH_4_ (35–38). Motivated by observations of inefficient methane generation in a ferruginous, sulfate-poor lake ostensibly representative of Precambrian conditions, biogeochemical models of low Precambrian methane have been proposed (39). However, ref. 40 found that such model behavior is dictated by arbitrary forcings and is not compatible with the rock record. In any case, hydrogenotrophic methanogenesis in the Archean water column could maintain substantial CH_4_ fluxes regardless of organic burial efficiency in sediments (35, 38, 39).

## Results

### The Case for Methane as a Biosignature.

Methane has been highlighted as a potential biosignature gas because it has a short photochemical lifetime (less than ∼1 My) on habitable-zone, rocky planets orbiting solar-type stars. A short photochemical lifetime requires substantial replenishment fluxes to sustain large atmospheric abundances. Methane is removed from an atmosphere photochemically in two ways, depending on the concentration of CO_2_ relative to CH_4_ and the presence of other oxidants (41). In the case where CO_2_ is significantly more abundant, CH_4_ is destroyed by oxidants and is converted to CO_2_ (see *SI Appendix*, section 3 for additional reactions):

[3] $$CH_{4}+\mathrm{h\nu}\to CH_{3}+H(↑\mathrm{space})$$

or

[4] $$CH_{4}+\mathrm{OH}\to CH_{3}+H_{2}O$$

and, subsequently,

[5] $$CH_{3}+O\to H_{2}\mathrm{CO}+H(↑\mathrm{space}).$$

The C in H_2_CO is further oxidized to CO_2_. The H produced can then be lost to space, thereby irreversibly destroying CH_4_. Note that OH and O are byproducts of H_2_O and CO_2_ photolysis; an O_2_-rich atmosphere is not required for rapid CH_4_ destruction, although it does decrease the CH_4_ lifetime.

For the case where CH_4_ is more abundant than CO_2_, CH_4_ polymerizes to aerosols, which fall to the ground and remove the atmospheric CH_4_ (see *SI Appendix*, section 3 for sequence of reactions). If temperatures are high enough in the lower atmosphere, these aerosols could break down and release CH_4_ back into the atmosphere. In addition, surface deposition and subsequent thermal decomposition in the subsurface could release methane back into the atmosphere. However, some portion of the hydrogen produced by methane photolysis is lost to space, and so, without H_2_ replenishment, the C:H ratio of condensate material will rise such that the methane is irreversibly lost.

The short atmospheric lifetime of terrestrial planet methane can be quantified. Using the photochemical model PhotochemPy adapted from the Atmos code (42) and created by N. Wogan (43) (*SI Appendix*, section 6A), we explore the stability of atmospheric CH_4_ for an Archean Earth-like planet (i.e., N_2_-CO_2_-CH_4_) orbiting a 2.7-Ga Sun-like star. Every calculation conserves redox. Consistent with previous studies (7, 13, 44, 45), we find that for atmospheric CH_4_ mixing ratios greater than ∼10^−3^ to be stable against photochemistry requires replenishing CH_4_ surface fluxes that are larger than Earth’s current biological flux (*SI Appendix*, Fig. S1). If a planet is orbiting a different stellar-type host star, it will be necessary to recalculate the threshold for biological methane surface fluxes. For example, planets orbiting M-stars tend to have lower near-UV radiation compared to Sun-like stars, which reduces the OH produced by H_2_O photolysis, permitting higher atmospheric CH_4_ concentrations (46). Ultimately, however, a terrestrial planet atmosphere that is rich in CH_4_ cannot persist unless there is a significant replenishment source flux, making it an intriguing candidate for further investigation.

#### Methane biosignatures and chemical disequilibrium.

The methane biosignature case is strengthened if its presence in the atmosphere is accompanied by that of a strongly oxidizing companion gas such as CO_2_ or O_2_/O_3_. This is because it is difficult to explain abundant methane if a terrestrial planet’s atmospheric redox state is sufficiently oxidized such that the thermodynamically stable form of carbon is not CH_4_. Methane in O_2_-rich atmospheres requires large replenishment fluxes because CH_4_ and O_2_ are kinetically unstable and out of thermodynamic equilibrium (47, 48). The kinetic lifetime of methane in O_2_-rich atmospheres is ∼10 y (44) due to the following net reaction, which is the end result of reactions **3** to **5** above after the H_2_CO has been further oxidized to CO_2_:

[6] $$CH_{4}+O_{2}\to CO_{2}+H_{2}O.$$

Another important thermodynamic disequilibrium is that between CH_4_ and CO_2_, which was present on the Archean Earth prior to the rise of O_2_. Specifically, CH_4_, CO_2_, N_2_, and liquid H_2_O coexisted out of equilibrium on the early Earth due to the replenishment of CH_4_ by life (14). In a weakly reduced Archean atmosphere, CH_4_’s lifetime would have been short (up to ∼2,000 to 20,000 y) compared to geologic timescales (49, 50). This short kinetic lifetime of methane does not depend on this thermodynamic disequilibrium with CO_2_; methane has a short photochemical lifetime in high mean-molecular-weight atmospheres regardless of whether or not CO_2_ is present in abundance. However, the thermodynamic disequilibrium is of fundamental importance for the discussion of abiotic methane that follows. Crucially, CH_4_ and CO_2_ are at opposite ends of the redox spectrum for carbon, separated by eight electrons. This has implications for how both species can be produced via abiotic planetary interior processes, which we explore subsequently; see the discussion of CO below. On the basis of both this thermodynamic disequilibrium and methane’s short photochemical lifetime, Krissansen-Totton et al. (14) argued that detecting both abundant CH_4_ and CO_2_ in a habitable-zone rocky exoplanet may be a biosignature and, if CH_4_’s mixing ratio is greater than ∼0.001, the methane is probably biogenic because it is challenging for abiotic sources to sustain large methane fluxes in anoxic atmospheres, similar to the findings of ref. 6.

#### CO antibiosignatures and their relationship to CH4 biosignatures.

In the above scenario, the absence of significant atmospheric CO may strengthen the case for biogenic CH_4_ since 1) microbial life readily consumes CO, a source of free energy, and 2) many abiotic processes that produce CH_4_ also result in abundant CO (14, 51) (and see below on magmatic outgassing). Life on Earth metabolizes CO because oxidizing it with water yields free energy and because CO metabolism serves as a starting point for carbon fixation (52, 53). Multiple lines of evidence suggest that CO consumption could be a ubiquitous metabolic strategy given its ancient origin on Earth (32, 53–55) and because the required enzymes possess a variety of simple Ni-Fe, Mo, or Cu active sites, suggesting that they have evolved independently multiple times (53, 56, 57). However, the mere presence or absence of CO may not be an unambiguous discriminator between a CH_4_-producing biosphere and an uninhabited world. An inhabited planet may have CH_4_, CO_2_, and some CO in its atmosphere if life is unable to efficiently consume all of the CO (11, 37, 38). In this case, however, the CO/CH_4_ atmospheric ratio in terrestrial planets’ high mean-molecular-weight atmospheres could potentially be used as a diagnostic tool to distinguish anoxic, inhabited planets from lifeless worlds because the CO/CH_4_ atmospheric ratio reflects the fractional atmospheric free energy that has been exploited.

**Fig. 1.**
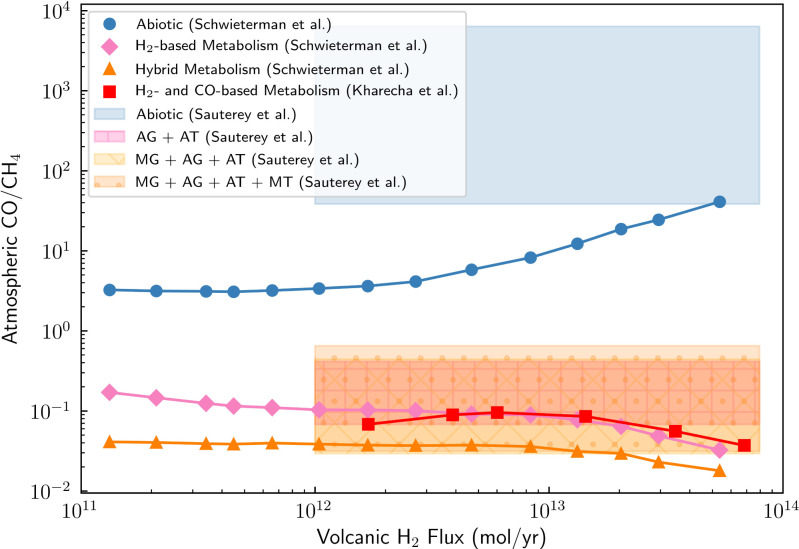
Atmospheric CO to CH_4_ ratio may help distinguish biogenic and abiotic methane. Shown is ratio of atmospheric CO to CH_4_ for abiotic worlds and those with biospheres as a function of volcanic H_2_ flux. The curves show the calculated atmospheric CO/CH_4_ as a function of volcanic H_2_ flux for abiotic worlds (blue circles), H_2_-based biospheres (includes H_2_-consuming anoxygenic photosynthesis, CO-consuming acetogenesis, organic matter fermentation, and acetotrophic methanogenesis) (pink diamonds), H_2_-based and Fe-based photosynthesis biospheres (i.e., “hybrid,” orange triangles) from ref. 37, and the methanogen–acetogen ecosystem and anoxygenic phototroph–acetogen ecosystem from ref. 35 (i.e., their cases 2 and 3) (red squares). The horizontal shaded regions correspond to the distributions of atmospheric CO/CH_4_ for abiotic worlds (blue) and those with methanogenic biospheres (pink, yellow, and orange) as a function of volcanic H_2_ flux calculated by ref. 38. The atmospheric CO/CH_4_ for abiotic worlds is predicted to be several orders of magnitude greater than that for inhabited worlds. Refs. 35, 37, and 38 found that low CO/CH_4_ atmospheric ratios (∼0.1) are a strong sign of methane-cycling biospheres for reducing planets orbiting Sun-like stars like Archean Earth, suggesting that atmospheric CO/CH_4_ is a good observable diagnostic tool to distinguish abiotic planets from those with anoxic biospheres. The light pink “+”-hatched region corresponds to an ecosystem with CO-based autotrophic acetogens (AG) and methanogenic acetotrophs (AT); the light orange “X”-hatched region corresponds to an ecosystem with H_2_-based methanogens (MG), AG, and AT; the orange “.”-hatched region corresponds to the most complex ecosystem consisting of MG, AG, AT, and anaerobic methanotrophy (MT) (38). All calculations assume a CO_2_-CH_4_-N_2_ bulk atmosphere.

Kharecha et al. (35), Schwieterman et al. (37), and Sauterey et al. (38) found that the atmospheric CO/CH_4_ ratio for abiotic worlds is predicted to be approximately two orders of magnitude larger than that for inhabited worlds that have anoxic biospheres over a wide range of volcanic H_2_ fluxes (Fig. 1). Note that we consider only the ecosystems from refs. 35 and 38 where both methanogenesis and CO consumption (acetogenesis plus acetotrophy) have evolved; if these conditions are not met, then larger CO/CH_4_ ratios are possible, but note the arguments for rapid emergence of CO consumption outlined above. While the atmospheric CO/CH_4_ ratio is likely an observable parameter that can be used to distinguish lifeless from inhabited, anoxic worlds, additional modeling is required to explore the possible range of CH_4_, CO_2_, and CO abundances for a wide variety of biospheres and uninhabited worlds around different host star types.

### Abiotic Sources of Methane.

**Fig. 2.**
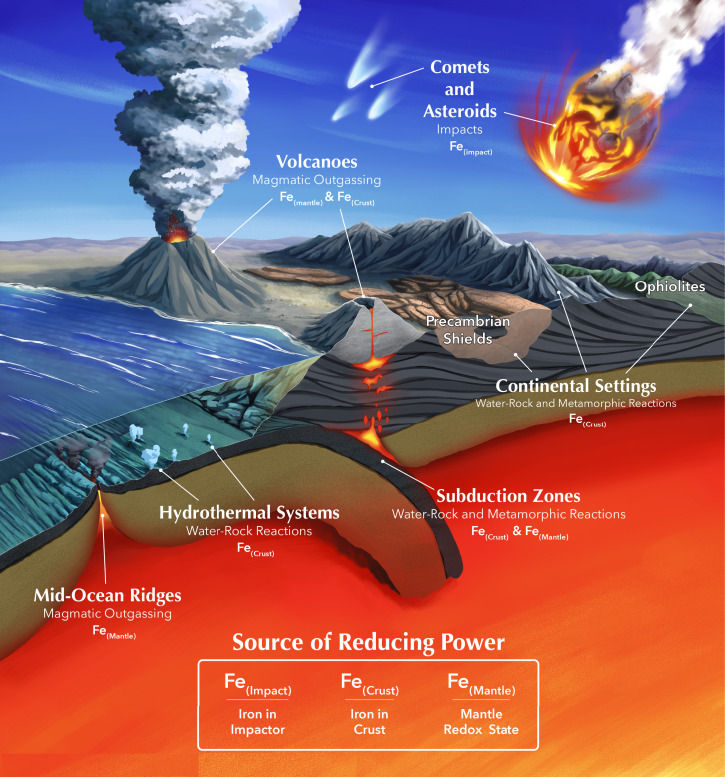
Summary of known abiotic sources of methane on Earth (copyright] 2022 Elena Hartley) (http://www.elabarts.com). In general, the abiotic sources of methane can be divided into three categories: high-temperature magmatic outgassing (volcanism), low-temperature water–rock and metamorphic reactions, and impacts. Currently, subaerial (submarine) volcanoes on Earth generate only ≤ 10 ^−3^ (∼10^−2^) Tmol/y of methane (see main text). Low-temperature water–rock reactions that generate methane occur at midocean ridges, deep-sea hydrothermal vents, subduction zones, and continental settings. Methane can also be generated by metamorphic reactions, particularly in subduction zones and continental settings such as ophiolites, orogenic massifs, and Precambrian shields. Both water–rock and metamorphic reactions can generate variable quantities of methane depending on the geochemical conditions, but, on Earth, methane fluxes are orders of magnitude smaller than biological sources. Finally, impacts or other exogeneous sources can generate methane. The impact flux was larger during earlier periods in Earth’s history, and such large impact fluxes are necessary to generate significant methane. A critical factor that influences the amount of methane that can be generated via all of these processes is the source of reducing power; in comparatively oxidizing surface environments with abundant CO_2_, a reductant is needed to reduce carbon to CH_4_. For magmatic outgassing, the reducing power ultimately comes from the mantle, with more reduced mantles outgassing more methane relative to CO_2_ and CO. For low-temperature water–rock and metamorphic reactions, the key source of reducing power is ferrous iron (Fe^2+^) in the crust, and in some cases the redox state of the mantle can also influence methane generation. For impact events, the metallic or ferrous iron that is delivered by the impactor serves as the source of reducing power.

While the vast majority of Earth’s atmospheric methane is produced biotically (28), there are various small abiotic sources of methane that could potentially be enhanced on other planets. Understanding plausible abiotic methane fluxes is necessary for discriminating methane biosignature false-positive scenarios from true signs of metabolism. These abiotic sources can be broadly divided into the following categories (Fig. 2): 1) volcanism and high-temperature magmatic processes, 2) low-temperature water–rock and metamorphic reactions, and 3) impact events.

#### Volcanism/high-temperature magmatic outgassing.

Volcanoes on Earth today do not outgas significant methane. Most subaerial volcanoes produce less than ∼10^−6^ Tmol CH_4_ per year (10, 58), and given the ∼1,500 active volcanoes on Earth today, the estimated global CH_4_ flux is <10^−3^ Tmol/y, much less than the current biogenic flux of 30 Tmol/y. Similarly, Schindler and Kasting (6) estimated the CH_4_ flux from submarine volcanism to be ∼10^−2^ Tmol/y. Although mud volcanoes, geological structures that transport clay rocks and sediment from Earth’s interior to the surface, can emit large amounts of methane and CO_2_ (59), the methane is largely thermogenic, ultimately deriving from organic matter produced by life (60). In principle, a terrestrial planet could abiotically emit methane through mud volcanoes given an abiotic source for the organic matter, such as hydrocarbon deposition from an organic haze. But that organic matter would need to be continuously replenished, and it is challenging for abiotic sources to provide the necessary replenishment (16, 42), especially under conditions sufficiently oxidizing to maintain a CO_2_-rich atmosphere.

Wogan et al. (11) investigated whether magmatic outgassing could produce genuinely abiotic CH_4_ fluxes on terrestrial planets with diverse compositions and surface conditions. They determined that volcanoes are unlikely to produce CH_4_ fluxes comparable to Earth’s biological flux because water has a high solubility in magma, which limits how much hydrogen (and therefore CH_4_) can outgas. Also, CH_4_ formation is thermodynamically favorable at temperatures lower than typical magma temperatures on Earth and at magma oxygen fugacities much more reduced than those expected for most terrestrial planets (11).

Could planets with significantly more reduced mantles and crusts produce high CH_4_ fluxes via magmatic outgassing? Mercury’s silicate interior has a low oxygen fugacity of ∼5 log_10_ units below the iron-wüstite (IW) redox buffer, and its crust is enriched in graphite, a crystalline form of carbon (61, 62). While Mercury’s small size and proximity to the Sun preclude the retention of an atmosphere, if there are large terrestrial exoplanets with similarly reducing interiors, then it is important to determine whether magmatic outgassing could produce CH_4_-rich atmospheres.

**Fig. 3.**
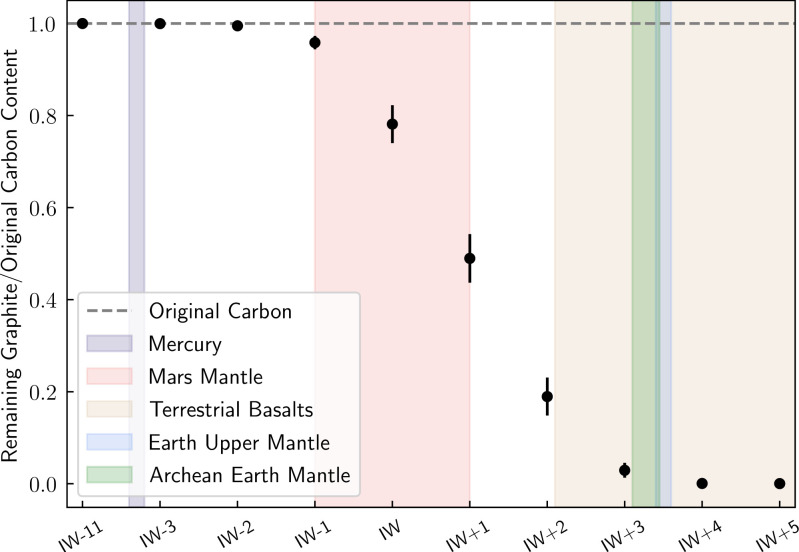
Most carbon partitions into graphite under reducing conditions and so cannot degas as CH_4_. Shown is the ratio of the amount of remaining graphite to the original carbon content as a function of oxygen fugacity. We used a batch-melting model to determine how volatiles would partition between the rock and melt over an ∼10-km deep column of newly produced crust with pressures from ∼0 to 0.5 GPa and temperatures from 1,400 to 1,445 K (*SI Appendix*, section 6B). For each oxygen fugacity, we ran a Monte Carlo simulation varying the input parameters, including CO_2_ and H_2_O mass fractions in the mantle source rock, the fraction of source material that is melted during emplacement, and the planetary melt production rate. The average ratio of remaining graphite to initial carbon content from the Monte Carlo simulation is shown with the uncertainty reported as the 95% confidence interval. The horizontal dashed line (y = 1) illustrates the original amount of carbon, and ratios that fall on this line have all of the original carbon stable as graphite. The shaded vertical regions show the estimated oxygen fugacities of Mercury’s lavas (61), the Martian mantle (65), terrestrial basalts (66), Earth’s upper mantle (67), and Archean Earth’s mantle (68) for reference.

Following the melting and volatile partitioning methods used in ref. 63, we applied a batch melting model, which assumes a partial melt is in equilibrium with the source rock before it rises to the surface, to determine the partitioning of volatiles from the rock to the melt (*SI Appendix*, section 6B). We assume the partitioning of carbon between the melt and solid phases is controlled by oxygen fugacity-dependent graphite saturation. For the top ∼10 km of crust (pressures from ∼0 to 0.5 GPa and solidus temperatures from ∼1,400 to 1,445 K), we ran a Monte Carlo simulation to explore a range of source rock CO_2_ and H_2_O concentrations, melt fractions, and planetary melt production volumes with oxygen fugacities from IW–11 to IW+5 (*SI Appendix*, Table S1). We find that for very reduced melts at or below IW–2, essentially all of the carbon (>99%) will precipitate as graphite during partial melting, so there is negligible carbon available for gaseous phases (Fig. 3 and *SI Appendix*, Fig. S2), consistent with observations of Mercury’s graphite-enriched crust (64). Rocky exoplanets with ultrareduced magma compositions are unlikely to outgas significant quantities of CH_4_ due to graphite saturation, although more experiments are needed to confirm reduced magmas’ outgassing compositions.

In the rare cases where volcanoes could produce biogenic levels of CH_4_ assuming magma production rates larger (>10 times) than those on Earth today, they would also outgas significant amounts of carbon monoxide (CO) gas (11). As described above, the atmospheric CO/CH_4_ ratio could be used to distinguish between abiotic (outgassed) and biotic scenarios (11, 37). Ultimately, high-temperature magmatic outgassing, such as through volcanism, is unlikely to produce atmospheric CH_4_ fluxes similar to those produced by biology on Earth.

#### Low-temperature water–rock reactions and metamorphic reactions.

The reliability of methane as a biosignature on habitable planets depends upon the tendency of low-temperature (below solidus) systems to generate methane via abiotic reactions. Under oxidizing planetary conditions conducive to CO_2_ degassing, low-temperature CH_4_ production is ultimately limited by the supply of reducing power in the form of ferrous iron (Fe^2+^) in newly produced crust. One of the most frequently discussed processes for methane production is serpentinization, through which iron-bearing minerals are altered by hydration to produce H_2_ via the oxidation of Fe ^2+^ by water (10, 69, 70):

[7] $$3\mathrm{FeO}+H_{2}O\to Fe_{3}O_{4}+H_{2}.$$

Subsequently, H_2_ can react with oxidized forms of carbon to produce CH_4_ by Fischer–Tropsch-type (FTT) reactions:

[8] $$4H_{2}+CO_{2}\to CH_{4}+2H_{2}O.$$

Metamorphic reactions may also produce CH_4_ via iron oxidation. For example, Fe-bearing carbonates can decompose when metamorphosed and react with water to form CH_4_ (71):

[9] $$\begin{array}{l} 3\mathrm{FeC}O_{3}+H_{2}O\to Fe_{3}O_{4}+CO_{2}+\mathrm{CO}+CH_{4}\\ \;\;\;\;\;\;+\text{Hydrocarbons}. \end{array}$$

Experimental methane and hydrocarbon yields via such reactions are typically very low compared to that of CO_2_ (72).

Experimental, observational, and theoretical approaches have been taken to determine the efficiency of hydrothermal and metamorphic processes and their corresponding abiotic CH_4_ production fluxes on Earth and how they may apply in other planetary environments. Various geological settings are potentially conducive to CH_4_ generation, including midocean ridges, subduction zones, and continental settings. For example, Keir (73) and Cannat et al. (74) investigated the concentrations of CH_4_ produced by serpentinization at midocean ridges and both found global abiotic CH_4_ fluxes to be about three orders of magnitude smaller than the global biogenic CH_4_ flux. Combining observational and theoretical approaches, Catling and Kasting (75) estimated abiotic hydrothermal CH_4_ fluxes from both axial and off-axis vents ranging from 0.015 to 0.03 Tmol/y. In addition, Guzmán-Marmolejo et al. (7) and Kasting (8) determined abiotic CH_4_ fluxes from hydrothermal systems ranging from 0.1 to 0.4 Tmol/y at present, and Kasting (8) found that this flux may potentially have been larger during the Hadean, ∼1.5 Tmol/y, but this is still over an order of magnitude smaller than the current biogenic flux. Brovarone et al. (76) and Fiebig et al. (77) estimated abiotic hydrothermal CH_4_ fluxes at subduction zones, finding modern fluxes of ∼10^−2^ Tmol/y similar to the above estimates. In continental settings, abiotic methane has been reported in low-temperature environments such as orogenic massifs and intrusions, seeps, crystalline shields, and ophiolites, with serpentinization of (iron-bearing) peridotites being the major source of methane in these settings (Fig. 2) (78). However, the amount of abiotic methane generated in continental settings is several orders of magnitude smaller than the biogenic flux (78–82).

Experimental studies on abiotic CH_4_ production via water–rock and metamorphic reactions have also been conducted. The availability of H_2_, the amount of excess aqueous carbonates, and the presence of mineral catalysts can greatly affect the amount of CH_4_ generated experimentally (83, 84). While Oze et al. (84) and Neubeck et al. (85) found that CH_4_ production by serpentinization is enhanced by the presence of mineral catalysts (e.g., chromite, magnetite, and awaruite), McCollom (71) cautions that these experimental studies did not quantify their organic contamination. McCollom (86) used isotopic labeling to differentiate CH_4_ produced by serpentinization from background sources. McCollom (86) found abiotic CH_4_ formation via serpentinization to be extremely limited, with most of the experimentally generated CH_4_ deriving from background sources. While iron oxidation and FTT-type reactions (or their metamorphic equivalents) are the most commonly discussed mechanisms for large abiotic fluxes on terrestrial planets, other possible mechanisms for reducing carbon include direct carbonate methanation and hydration of graphite-carbonate–bearing rocks, but they are also unlikely to generate false-positive scenarios (*SI Appendix*, section 2).

**Fig. 4.**
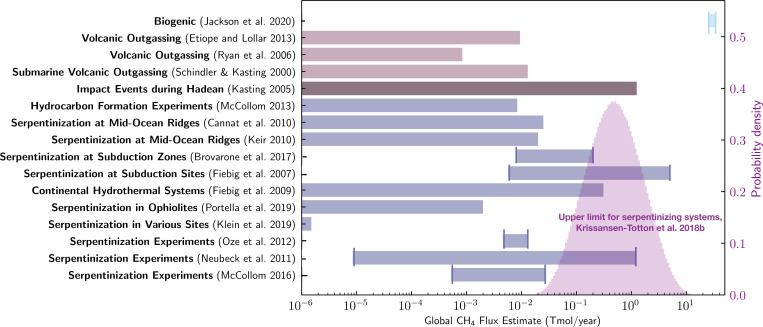
Summary of known abiotic CH_4_ sources with their estimated global CH_4_ flux values compared to Earth’s current biogenic CH_4_ flux. As in *SI Appendix*, Table S2, for each abiotic source considered, we present those sources for which we can estimate global CH_4_ flux values from a given reference. In the cases where there are multiple global CH_4_ flux estimates for a given reference of an abiotic source, we show the maximum and minimum CH_4_ flux estimates by the vertical lines (6, 8, 10, 14, 28, 58, 71, 73, 74, 76, 77, 79, 81, 82, 84–86). The transparent purple probability distribution for the maximum abiotic CH_4_ flux from serpentinization is from ref. 14, and the right-hand *y* axis shows the probability density of this distribution. None of the abiotic sources considered have estimated global CH_4_ fluxes that are similar to or exceed Earth’s modern biogenic CH_4_ flux. In fact, most of the abiotic sources have predicted global CH_4_ fluxes that are at least an order of magnitude less than Earth’s biogenic CH_4_ flux. We do not show the flux estimates that exceed the iron supply because such extremely large fluxes are based on experimental results for which there are issues with organic contamination (main text).

The critical limitation of hydrothermal CH_4_ production is the supply of Fe^2+^ and the efficiency with which iron can be oxidized to generate CH_4_. The availability of iron and the efficiency of its oxidation on a planetary scale depend on a range of geological and geochemical processes that operate across disparate spatial and temporal scales. Tectonic regime, mineral catalysis, volatile inventories, surface climate, and crustal composition and permeability/porosity all potentially modulate the efficiency and extent of crustal hydration. To investigate this process’s limitations, Krissansen-Totton et al. (14) estimated the maximum CH_4_ flux generated via serpentinization by exploring plausible ranges of parameters including crustal production rate, the fraction of FeO in fresh crust, the maximum fractional conversion of FeO to H_2_ via serpentinization, and the maximum fractional conversion of H_2_ to CH_4_ via FTT reactions. Producing a probability distribution for the maximum abiotic CH_4_ flux, they found that Earth-like biological CH_4_ fluxes are at least an order of magnitude larger than plausible abiotic fluxes from serpentinization, consistent with the findings of the studies discussed above (14) (Fig. 4).

Ultimately, abiotic CH_4_ generation via low-temperature water–rock or metamorphic reactions is unlikely to produce atmospheric CH_4_ fluxes comparable to modern biotic fluxes in combination with atmospheric CO_2_ (*SI Appendix*, Table S2 and Fig. 4). In fact, all CH_4_ flux extrapolations from low-temperature system studies discussed above are consistent with the maximum abiotic flux estimates in ref. 14. Nevertheless, the possible parameter space for crustal methane production is vast, and work remains to be done to determine whether unfamiliar environmental conditions may exist on other planets that could produce a false-positive signal. For example, Fe-enriched olivine may be more common compositions for the mantles of other rocky planets compared to the Mg-rich olivine characteristic of Earth’s mantle. McCollom et al. (87) determined that serpentinization of Fe-rich olivine can generate significantly more H_2_ compared to that of Mg-rich olivine (by a factor of ∼2 to 10) (87). Another source of uncertainty is what catalysts might be available in natural settings. At temperatures ≤600 K, in gas mixtures with CO_2_ and H_2_, CH_4_ is thermodynamically preferred, but the reaction is kinetically inhibited and will proceed only if catalyzed. Future investigations could seek to develop coupled geochemical evolution models of a planet’s mantle and crust that can self-consistently predict CH_4_, CO_2_, and CO fluxes from high-temperature magmatic processes and low-temperature hydrothermal and metamorphic systems, such that the contextual clues of abiotic methane can be explored for different compositional assumptions.

#### Impacts.

The solar system terrestrial planets likely experienced a late-accreting veneer from impacts of comets and asteroids prior to 3.8 Ga (88). Impact events are plausible abiotic sources that can generate methane in two ways: 1) After a cometary impactor hits a planet, it vaporizes, and in the cooling impactor, some of the molecules delivered by the impactor may react to form CH_4_ (89); and 2) large asteroid impactors could deliver a reducing power (i.e., iron) and vaporize a planet’s surface ocean, causing a steam atmosphere to form, and CH_4_ may form in such a cooling steam atmosphere (41). To generate significant methane, impact events require either a large, constant flux of impactors (case 1) or a transient postimpact atmosphere from a giant impact event (case 2).

For case 1, Kress and McKay (89) and Kasting (8) modeled CH_4_ formation from volatile-rich impactors. Ref. 89 found that a 1-km comet can generate 0.6 Tmol of atmospheric CH_4_ per impact event, and ref. 8 estimated that the global CH_4_ impact flux during the Hadean was ∼1.25 Tmol/y. However, it is unknown whether condensing dust from cometary impactors has effective catalytic properties to enable CH_4_ generation. Recent theoretical and experimental work investigated the outgassing compositions of chondritic materials that may represent cometary impactors and found that there are small to negligible amounts of outgassed CH_4_ from some of the most volatile-rich chondrites (i.e., CM chondrites) (90, 91).

For case 2, Zahnle et al. (41) showed that a transient reducing atmosphere (rich in CH_4_, H_2_, and NH_3_) could have been generated on the early Earth by large asteroid impacts during the late-accreting veneer. Such giant impacts would produce methane since they delivered metallic iron, a significant reducing power, to the surface (41). The iron could react with Earth’s existing H_2_O to produce H_2_ and FeO, which would subsequently react with atmospheric CO_2_ or CO to produce CH_4_. The amount of methane that could form depends on the amount of carbon available prior to the impact, how much iron the impactor delivers, how much of that iron reacts with the atmosphere, and the presence of catalysts that can reduce the quench temperature so methane is thermodynamically stable (41). A possible false-positive scenario is one in which a giant impact event could produce a transient atmosphere with abundant CH_4_ and CO_2_ but low CO. However, calculations of transient impact-generated atmospheres of ref. 41 suggest that such false-positive scenarios are unlikely to be long lived for significant portions of geologic time and would be accompanied by H_2_-dominated atmospheres (e.g., figures 7, 8, and 12 in ref. 41).

### Methane Beyond Earth: Mars and Temperate Exo-Titans.

Methane exists in other locations besides Earth throughout the solar system, including in the atmospheres of the outer planets and in comets (92). While super-Earths and sub-Neptune planets do not exist in our solar system, they are common among other planetary systems, and future studies could determine the surface pressures necessary for these planets to sustain methane via thermochemical recombination, without the need for a significant surface flux (*SI Appendix*, section 5). For example, if atmospheric H_2_ is abundant, then CH_4_ will efficiently recombine after photolysis, which dramatically increases the CH_4_ lifetime (*SI Appendix*, section 3). As the focus of this study is on terrestrial planets, this section discusses atmospheric methane sources in other terrestrial worlds, in particular Mars and temperate Titan-like exoplanets (exo-Titans).

#### Mars.

The presence of methane on Mars is debated, with claims of detections at the ∼10 to 60 ppbv level that are highly variable in time and space by the European Space Agency’s (ESA) Mars Express, NASA’s Curiosity rover, and ground-based observations (52, 93–95). However, the most recent and most sensitive measurements by the ESA-Roscosmos ExoMars Trace Gas Orbiter did not detect any significant methane over all observed latitudes and reported an upper limit of ∼20 ppt methane for altitudes above a few kilometers, several orders of magnitude lower than all previous purported CH_4_ detections (96). Regardless, methane detections of a few parts per billion to tens of parts per billion are much lower than the terrestrial exoplanet thresholds for biogenic CH_4_ considered in this study. There are a variety of plausible abiotic explanations for methane on Mars, including water–rock reactions, the release of clathrates, and degradation of organic matter.

#### Temperate exo-Titans.

Methane exists (at ∼1 to 5%) in the N_2_-rich atmosphere of Saturn’s largest moon Titan (97). Photochemical models predict that the current CH_4_ in Titan’s atmosphere would be destroyed in ∼30 My unless there is a mechanism that resupplies CH_4_ to the atmosphere (98, 99). Possible mechanisms for Titan’s CH_4_ resupply include its subsurface ocean, CH_4_ clathrate hydrates in the crust, liquid hydrocarbons in the subsurface, or outgassing from the interior (100). While life has been suggested as a possible explanation (101), the absence of conventionally habitable surface conditions makes geochemical processes more attractive explanations.

**Fig. 5.**
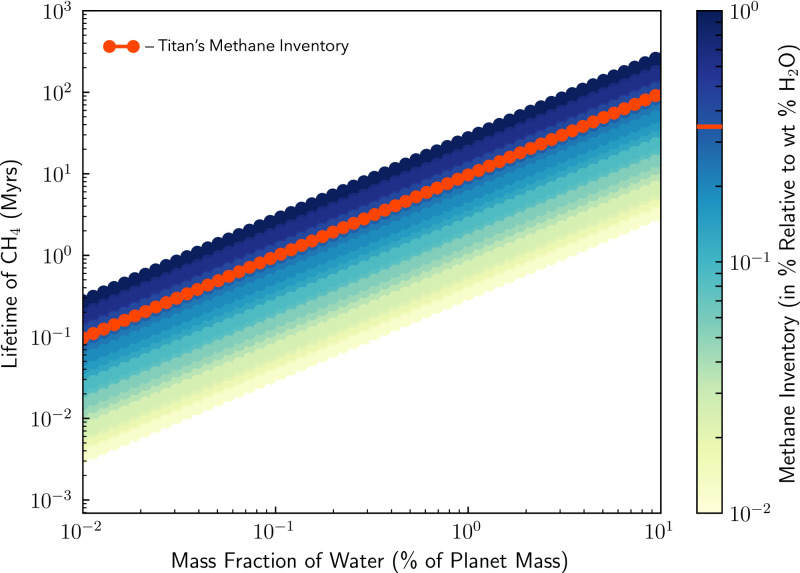
The photochemical lifetime of methane biosignature false positives produced by melting volatile-rich Titan analogs is short. Shown is the estimated lifetime of atmospheric methane as a function of the planet’s water mass and initial methane volatile inventory. Assuming methane’s escape rate is diffusion limited and that its steady-state mixing ratio is 10%, we varied the initial methane volatile inventory (drawing values from a uniform distribution from 0.01 to 1.0% relative to weight % water, represented by the color bar) and the mass fraction of the planet’s water (exploring values from 0.01 to 10% of the mass of the planet, assuming an Earth-mass planet) and calculated the estimated lifetime for methane in the atmosphere (*SI Appendix*, section 6D). The red curve represents Titan’s methane inventory (∼0.35%) (102). For planets with Titan-like methane inventories and water mass fractions that are 1% (10%) of the planet’s mass, the lifetime of atmospheric methane will be ∼10 My (∼100 My).

Whatever the source of Titan’s methane, temperate Titan-like exoplanets are unlikely to produce a CH_4_ + CO_2_ biosignature false positive. We estimate the atmospheric CH_4_ lifetime for an Earth-sized exoplanet with a Titan-like volatile inventory that migrates to the habitable zone where all surface ice melts (see *SI Appendix*, section 6D for a scenario where ice remains). Given initial CH_4_ and CO_2_ reservoirs relative to H_2_O based on Titan’s volatile inventory (102), we neglect oxidation via OH to be conservative and calculate the loss of CH_4_ via diffusion-limited hydrogen escape (103). We assume that the atmospheric mixing ratio of CH_4_ is 10%, which is conservative given the respective solubilities of CH_4_ and CO_2_ and plausible background N_2_ inventories (*SI Appendix*, section 6D). We find that for planets with water mass fractions that are <1.0 wt% of the planet’s mass, the atmospheric CH_4_ lifetime is short at habitable-zone separations (less than ∼10 My) (Fig. 5). If the water mass fraction is ∼10 wt% of the planet’s mass, then atmospheric CH_4_ may last for longer periods of time (∼100 My), but even so the duration is much shorter than typical stellar ages. In any case, it will likely be possible to identify planets with such large water inventories via their low densities. Whether hydrogen’s removal timescale could be dramatically lengthened via low loss rates or other large hydrogen reservoirs (while maintaining a CO_2_-rich atmosphere) is a promising topic for future computational studies.

## Discussion

### Toward Procedures to Identify Methane Biosignatures.

Any procedure for observationally identifying methane biosignatures must take into account the broader planetary and astrophysical context and will be dictated by the capabilities of the available instruments. Major steps might include the following: 1) detecting a terrestrial planet within the habitable zone of its host star and characterizing its bulk properties (e.g., mass, radius, orbital properties); 2) measuring its atmospheric composition, namely the abundances of CH_4_, CO_2_, CO, H_2_O, and H_2_ and confirming that the atmosphere is anoxic; and 3) identifying possible false positives and combining this information with observational data on the planet’s broader context to determine the likelihood of abiotic vs. biotic sources of methane (*SI Appendix*, Fig. S3). It is important that the host star is well characterized (i.e., UV radiation and stellar activity) to understand the planet’s photochemical environment. Identifying the presence of liquid water on the surface of a planet would suggest a particularly compelling target since it is a likely requirement for life.

Constraining the atmospheric abundances of CH_4_, CO_2_, and CO and confirming that the atmosphere is not H_2_ dominated is essential for determining whether the planet’s atmosphere is indicative of the presence of a biosphere. Terrestrial planets with high mean-molecular-weight atmospheres are better candidates to search for methane biosignatures because in such atmospheres, the CH_4_ lifetime will be very short without a significant replenishment source. In addition, confirming that the planet’s atmosphere is anoxic is necessary to distinguish a false-positive case for an anoxic planet with abundant atmospheric CH_4_, CO_2_, and CO from an oxic planet with an oxygen-based biosphere that has atmospheric CH_4_, CO_2_, CO, and O_2_ (37). With these abundances constrained, a photochemical model can infer the surface fluxes of the atmospheric constituents. Indications that these surface fluxes may be consistent with a biosphere include large implied CH_4_ fluxes coexisting with atmospheric CO_2_ but comparatively low CO abundances.

Even if the surface fluxes are consistent with a biosphere, it is necessary to identify all possible false positives including magmatic outgassing from a reduced mantle (Fig. 3), water–rock and metamorphic reactions (Fig. 4), large impact fluxes, and large volatile inventories (Fig. 5). The viability of detecting methane biosignatures depends on our knowledge of abiotic methane sources and their production rates. One of the most outstanding uncertainties is an incomplete understanding of plausible abiotic methane production on a planetary scale via water–rock and metamorphic reactions. If a planet has an atmospheric composition consistent with a methanogenic biosphere but false positives cannot be entirely ruled out, it will be necessary to search for corroborating evidence such as additional biosignature gases [e.g., methyl chloride (46), organosulfur compounds (104)], signs of atmospheric seasonality, and reflectance signatures from pigmented surface organisms (105, 106) (*SI Appendix*, Fig. S3). Ultimately, definitively detecting the presence of methane biosignatures on a terrestrial exoplanet will require taking into account the entire planetary and astrophysical context, characterizing the planet’s atmospheric composition, investigating all potential false-positive scenarios, and likely searching for supporting evidence.

### Detectability Prospects.

Prospects for detecting biogenic levels of methane in terrestrial exoplanet atmospheres in the near future with JWST are promising (17, 24, 25, 27). However, it may be challenging to obtain sufficient observational data on the planetary context to confirm the presence of methane biosignatures and rule out false positives. Although JWST may be able to detect CO_2_, it will provide only crude constraints on CO abundances (17, 27). Ref. 27 determined that JWST could place upper bounds on CO abundances in ∼10 transits and constrain the CO/CH_4_ ratio with more transits for an Archean Earth-like TRAPPIST-1e (27). Ref. 17 confirms that JWST will likely be able to crudely constrain the CO/CH_4_ ratio and notes that CO constraints will be possible with high-resolution spectroscopy measurements with extremely large telescopes (ELTs). If biospheres are dominated by oxygenic photosynthesis, they may produce large CO fluxes through biomass burning (37). Therefore, to distinguish an anoxic, lifeless world with abundant atmospheric CH_4_, CO_2_, and CO from an oxic, inhabited planet with CH_4_, CO_2_, CO, and O_2_ requires observations that can detect or rule out the presence of atmospheric O_2_/O_3_, which will be challenging with JWST (37). In addition, JWST will not be able to detect water vapor with transit observations due to water cloud condensation nor constrain surface properties, so it will not be able to fully assess habitability (107, 108). Nevertheless, if JWST detects significant CH_4_ and CO_2_ and places some constraints on the CO/CH_4_ ratio in a terrestrial exoplanet’s atmosphere, such a discovery would certainly motivate observations with future instruments.

Looking ahead, ground-based ELTs will help characterize terrestrial exoplanets and their biosignatures (109). Ref. 26 determined that for a cloud-free, low-CO_2_ TRAPPIST-1e atmosphere, a mere 10 ppm CH_4_ is likely detectable with high-resolution transit spectroscopy with the European ELT in less than ∼30 transits, and CO detections may be possible with ∼40 transits (26). In addition, the Astro2020 Decadal Survey recommended an ∼6m infrared/optical/UV space telescope to characterize the atmospheres of dozens of habitable-zone terrestrial exoplanets, including detecting methane (5, 110). Identifying methane biosignatures will require not only detecting and constraining the atmospheric abundances of CH_4_, CO_2_, and CO, but also using a combination of observational tools to comprehensively characterize the broader planetary context.

## Conclusions

With the upcoming technological advancements in exoplanet observations enabling the characterization of potentially habitable exoplanets, it is important to consider possible biosignature gases and the sources of false-positive detections. This is particularly urgent for methane since biogenic methane is likely detectable for some terrestrial exoplanets with JWST. The case for methane as a biosignature stems from the fact that photochemistry of terrestrial planet atmospheres implies that large CH_4_ surface fluxes are required to sustain high levels of atmospheric methane. Although a variety of abiotic mechanisms could, under diverse planetary environments, replenish atmospheric methane, we find that it is challenging for such sources to produce abiotic CH_4_ fluxes comparable to Earth’s biogenic flux without also generating observable contextual clues that would signify a false positive. For example, we investigated whether planets with very reduced mantles and crusts can generate large methane fluxes via magmatic outgassing and assessed the existing literature on low-temperature water–rock and metamorphic reactions and, where possible, determined their maximum global abiotic methane fluxes. In every case, abiotic processes cannot easily produce atmospheres rich in both CH_4_ and CO_2_ with negligible CO due to the strong redox disequilibrium between CO_2_ and CH_4_ and the fact that CO is expected to be readily consumed by life. We also explored whether habitable-zone exoplanets that have large volatile inventories like Titan could have long lifetimes of atmospheric methane. We found that, for Earth-mass planets with water mass fractions that are less than ∼1% of the planet’s mass, the lifetime of atmospheric methane is less than ∼10 My, and observational tools can likely distinguish planets with larger water mass fractions from those with terrestrial densities.

Clearly, the mere detection of methane in an exoplanet’s atmosphere is not sufficient evidence to indicate the presence of life given the variety of abiotic methane-production mechanisms. Instead, the entire planetary and astrophysical context must be taken into account to interpret atmospheric methane. *SI Appendix*, Fig. S3 illustrates a tentative procedure for identifying methane biosignatures in the atmospheres of habitable terrestrial exoplanets. Ultimately, methane is more likely to be biogenic on a habitable-zone planet when 1) planet bulk density is terrestrial (no large surface volatile reservoirs), the atmosphere has a high mean molecular weight and is anoxic, and the host star is old; 2) the atmospheric CH_4_ abundance is high, with implied surface replenishment fluxes exceeding what could plausibly be produced by known abiotic processes (∼10 Tmol/y); and 3) when atmospheric methane is accompanied by CO_2_ but comparatively little CO (or CO/CH_4_ < 1).

## Materials and Methods

We use the photochemical model PhotochemPy in *SI Appendix*, Fig. S1 (*SI Appendix*, section 6A). The calculations for determining how carbon partitions between different phases under various redox conditions for Fig. 3 follow the methods in ref. 63 and are discussed further in *SI Appendix*, section 6B. The global abiotic CH_4_ flux estimates in Fig. 4 are described in detail in *SI Appendix*, section 6C. For Fig. 5, we estimate the atmospheric CH_4_ lifetime for an Earth-mass terrestrial planet with different water mass fractions and Titan-like volatile inventories by assuming the escape flux of hydrogen is diffusion limited (*SI Appendix*, section 6D). The codes used for our analysis are available on GitHub at https://github.com/maggieapril3/MethaneBiosignature (*SI Appendix*, section 6).

## Supplementary Material

Supplementary File

## Data Availability

All data needed to evaluate the conclusions in this paper are present in this paper and/or in *SI Appendix*, *Materials and Methods*. PhotochemPy can be accessed at GitHub (https://github.com/Nicholaswogan/PhotochemPy). Python code data have been deposited in GitHub (https://github.com/maggieapril3/MethaneBiosignature) (111).
