# Supplementary material for: The case and context for atmospheric methane as an exoplanet biosignature
Source: Proc Natl Acad Sci U S A. 2022 Mar 30;119(14):e2117933119. doi: 10.1073/pnas.2117933119 (PMC9168929; doi:10.1073/pnas.2117933119)
Supplement: Supplementary File [file pnas.2117933119.sapp.pdf]

PNAS
www.pnas.org

Supplementary Information for

The Case and Context for Atmospheric Methane as an Exoplanet Biosignature

Maggie A. Thompson, Joshua Krissansen-Totton, Nicholas Wogan, Myriam Telus, Jonathan J. Fortney

Maggie A. Thompson.
E-mail: maapthom@ucsc.edu

This PDF file includes:

Supplementary text
Figs. S1 to S3 (not allowed for Brief Reports)
Tables S1 to S3 (not allowed for Brief Reports)
SI References

Supporting Information Text

1. Atmospheres with Abundant CH_4 and CO_2 in Chemical Equilibrium–Discussion of Woitke et al. 2021

Could there be a biosignature false-positive scenario in which abundant CH_4 and CO_2 (without CO) coexist in thermochemical equilibrium in a rocky planet's atmosphere? Woitke et al. 2021 (1) show that at temperatures below ~600 K, CO_2, CH_4, N_2, and H_2O could coexist in chemical equilibrium if aqueous species are neglected. However, Woitke et al. 2021 (1) did not consider photochemistry and its effects on the stability of these atmospheres. Photochemistry is essential when assessing the plausibility of proposed terrestrial planet atmospheres.

To illustrate how a large CH_4 surface flux would be required to sustain high levels of atmospheric CH_4, a series of photochemical models were run simulating terrestrial planet atmospheres. We used PhotochemPy, a photochemical model adapted from the Atmos code (2) and created by N. Wogan (https://github.com/Nicholaswogan/PhotochemPy), that uses a set of inputs (e.g., stellar flux, atmospheric temperature structure, chemical species and reactions) and then integrates the atmosphere forward in time until it reaches a photochemical steady state (see SI Section 6A). A series of models were generated assuming a planet with an initial atmospheric composition that is Archean Earth-like (i.e., N_2-CO_2-CH_4), orbiting a 2.7 Ga Sun-like star and explored a range of CO_2 and CH_4 surface mixing ratios from 0.1 to 0.5 and from 10^{-5} to 0.1, respectively.

Figure S1 illustrates that abundant atmospheric CH_4 in atmospheres containing CO_2, N_2, and H_2O, requires CH_4 surface fluxes similar to or greater than modern Earth's biogenic flux to balance photochemical destruction. Therefore, such an atmosphere will not be stable without a significant CH_4 replenishment source that likely exceeds Earth's modern biogenic flux. For example, in order to sustain high atmospheric CH_4 mixing ratios of ~0.1 along with significant amounts of CO_2, the required CH_4 surface fluxes are on the order of $\sim 3.7 \times 10^{12}$ molecules/cm^2/s (~1000 Tmol/year), corresponding to the yellow regions of Figure S1. Such a large CH_4 replenishment flux would be about 30 times larger than Earth's current biogenic flux (30 Tmol/year). Considering the global redox budget, such abundant atmospheric CH_4 requires that either the flux of reductants from Earth's interior is at least three orders of magnitude higher than Earth's modern hydrogen outgassing rate or that the H_2 escape rate is much less than the diffusion limit (3). In addition, the equilibrium calculations of (1) did not consider the formation of dissolved ammonium (NH_4^+) and bicarbonate (HCO_3^-), which are shown to be the equilibrium products of CO_2, CH_4, and N_2 in the presence of liquid water in (4). The thermochemical calculations of (1) could instead have relevance for deep sub-Neptune atmospheres.

2. Additional Water-Rock and Metamorphic Reactions and Key Unknowns

While iron oxidation and FTT-type reactions (or their metamorphic equivalents) are the most commonly discussed mechanisms for large abiotic fluxes on terrestrial planets (Figure 2), it is worth considering other possible mechanisms for reducing carbon. Direct carbonate methanation can produce CH_4 given an exogeneous supply of H_2 (5–8), as follows:

$$\mathrm{CaCO_3 + 4\,H_2 \longrightarrow CH_4 + Ca(OH)_2 + H_2O} \quad [1]$$

$$\mathrm{MgCO_3 + 4\,H_2 \longrightarrow CH_4 + Mg(OH)_2 + H_2O} \quad [2]$$

Here, the production of H_2 is likely to be limited by iron oxidation via water rock reactions, unless conditions are sufficiently reducing that H_2 rather than H_2O is the dominant H-bearing product from magmatic outgassing. In this scenario, however, simultaneously large fluxes and atmospheric concentrations of CO_2 are unlikely (Figure S2).

Hydration of graphite-carbonate bearing rocks can similarly generate CH_4 without the need for iron oxidation (9), in the following reaction:

$$\mathrm{Mg_3Si_4O_{10}(OH)_2(talc) + 3\,CaCO_3(calcite) + 6\,C(graphite) + 5\,H_2O \longrightarrow {}_3CaMg(CO_3)_2(dolomite) + 4\,SiO_2(quartz) + 3\,CH_4} \quad [3]$$

However, in the absence of biological organic matter, crustal compositions rich in graphite, require strongly reducing conditions (Figure 3). These are unlikely to coexist with high magmatic CO_2 fluxes, which require oxidizing conditions, without large magmatic fluxes of CO (Figure S2).

Some of the other fundamental unknowns with regards to water-rock and metamorphic reactions include the efficiency and extent of hydration reactions under different tectonic regimes, the importance of carbonate hydration in the presence of graphite in generating CH_4, the extent to which H_2 can directly react with carbonates to produce CH_4 under reducing melt conditions, and the extent to which heterogeneous surface environments could simultaneously produce high CH_4 and CO_2 fluxes.

3. Photochemical Destruction and Recombination Pathways for Methane

Methane is removed from an atmosphere photochemically in two ways, depending on the concentration of CO_2 relative to CH_4 and the presence of other oxidants (10). In the first case where CO_2 is more abundant, then CH_4 is destroyed by oxidants and ultimately is converted to CO_2, such as through the following reactions:

$$\mathrm{CH_4 + h\nu \longrightarrow CH_3 + H(\uparrow space)} \quad [4]$$

or

$$CH_4 + OH \longrightarrow CH_3 + H_2O \quad [5]$$

or

$$CH_4 + O^1D \longrightarrow OH + CH_3 \quad [6]$$

and, subsequently, either

$$CH_3 + O \longrightarrow H_2CO + H(\uparrow \text{space}) \quad [7]$$

or

$$CH_3 + O_2 \longrightarrow H_2CO + OH \quad [8]$$

The C in H_2CO is further oxidized to CO_2. The H produced can then be lost to space, thereby irreversibly destroying the CH_4. Note that OH, O^1D, O and O_2 are byproducts of H_2O and CO_2 photolysis; an atmosphere rich in molecular oxygen is not required for rapid CH_4 destruction (although it does decrease the CH_4 lifetime). The pathway involving OH and O_2 is the dominant destruction pathway in Earth's modern atmosphere, and all of these pathways were likely important for the Archean atmosphere (10).

For the second case where CH_4 is more abundant than CO_2, CH_4 polymerizes to aerosols, which fall to the ground and remove the atmospheric CH_4. The chemistry producing aerosols is complex, but this sequence of reactions (Equations 9-14) demonstrates the general process:

$$CH_4 + h\nu \longrightarrow {}^1CH_2 + H_2(\uparrow \text{space}) \quad [9]$$

$$^1CH_2 + N_2 \longrightarrow {}^3CH_2 + N_2 \quad [10]$$

$$^3CH_2 + {}^3CH_2 \longrightarrow C_2H_2 + H_2(\uparrow \text{space}) \quad [11]$$

$$C_2H_2 + h\nu \longrightarrow C_2H + H(\uparrow \text{space}) \quad [12]$$

$$C_2H_2 + C_2H \longrightarrow C_4H_2 + H(\uparrow \text{space}) \quad [13]$$

$$C_4H_2 + C_2H \longrightarrow \text{bigger hydrocarbons} \quad [14]$$

These hydrocarbons condense into aerosols that fall to the ground and thus remove CH_4 from the atmosphere. These aerosols could break down and release CH_4 back into the atmosphere or they could get buried and subducted into the planet. However, some portion of the hydrogen produced by methane photolysis will be lost to space, and so, without H_2 replenishment, the C:H ratio of condensate material will rise such that the methane is irreversibly lost.

Ultimately, the lifetime of atmospheric CH_4 is determined by the efficiency of the pathways outlined above. Atmospheric composition is an important determinant of that efficiency. For example, if H_2 is abundant, then CH_4 will efficiently recombine after photolysis via:

$$CH_3 + H + M \longrightarrow CH_4 + M \quad [15]$$

where M is an unspecified collision partner that carries away excess energy, which dramatically increases the CH_4 lifetime.

4. Gas Giant Planets

The giant planets in the Solar System contain abundant methane in their H_2-rich atmospheres due to accreting and processing primordial material from the solar nebula (11, 12). Methane in the atmospheres of giant planets can be replenished indefinitely because, although methane gets photodissociated in the upper atmosphere, hydrogen is never depleted via escape, and carbon and hydrogen can recombine deeper in the atmosphere where temperatures and pressures are high enough for methane production to be thermodynamically favorable and kinetically viable (13). Conversely, temperate terrestrial planets with high mean molecular weight atmospheres and surfaces do not have deep enough atmospheres to replenish methane without an additional source (abiotic or biotic). In terrestrial atmospheres without a replenishment source, methane is photodissociated and hydrogen is lost to space on short timescales (e.g., ~10's of thousands of years for ~1 bar atmospheres). However, if a H_2-rich atmosphere on a terrestrial planet exists, then the CH_4 lifetime would be long due to stabilizing reaction pathways like those that operate in giant planet atmospheres.

5. Super-Earths and Sub-Neptune Planets

Although the focus of this study is on methane biosignatures on terrestrial planets, it is important to consider methane in the atmospheres of super-Earths (planets with radii between 1 and ∼1.8 $R_\oplus$) and sub-Neptunes (radii larger than ∼1.8 $R_\oplus$ and less than ∼3.5 $R_\oplus$) (14). These planets are expected to span a diverse range in bulk compositions from rocky to gaseous, and therefore their atmospheres are also expected to have various compositions, from thick, H_2/He-rich primary atmospheres to thin secondary atmospheres comparable to that of terrestrial planets. Methane in sub-Neptune atmospheres would be unremarkable due to the possibility of thermodynamic recombination at depth. However, future studies could seek to determine the atmospheric pressure necessary for a planet to sustain methane via thermodynamic recombination against photodissociation. Observational methods to distinguish super-Earths from sub-Neptunes are also relevant for excluding deep atmosphere replenishment as a source of methane (15).

When searching for methane in the atmospheres of super-Earths and sub-Neptunes, as is the case for terrestrial planets, it is also important to understand the effects of the host star. For example, M dwarfs tend to be more active longer into their life cycles and have stronger far-UV and weaker near-UV emissions compared to solar-type stars, making it essential to determine how such host stars may impact such an exoplanet's atmosphere (e.g., photochemistry). Future work should aim to couple geochemical and photochemical models to better understand how cooler host stars can affect a planet's atmosphere.

6. Materials and Methods

All codes used in this study will be made publicly available upon publication at https://github.com/maggieapril3.

A. Photochemical Model: PhotochemPy. We use the PhotochemPy photochemical model in Figure S1 to illustrate the methane fluxes required to sustain atmospheric methane in various atmospheres. PhotochemPy is a descendant of the Photochem model contained in the Atmos modeling suite, which was originally developed by Jim Kasting and Kevin Zahnle (16) and has since been developed by many of their students and colleagues. Appendix B in (17) contains an in-depth description of the main equations solved in the Photochem model. The physics and chemistry in PhotochemPy are very similar to the physics and chemistry in the version of Atmos used in (2). The main exception is that we have updated several reactions, and water photolysis cross sections following (18). However, PhotochemPy deserves a distinct name because it is a modern Fortran rewrite of the original Fortran 77 code. Additionally, PhotochemPy uses Numpy F2PY (19) to generate a Python wrapper to the compiled Fortran library.

B. Carbon Partitioning and Magmatic Outgassing Calculations. For Figure 3 and Figure S2, to calculate how carbon partitions between different phases under various redox conditions for ∼10 km of crust (pressures from ∼0-0.5 GPa and solidus temperatures from ∼1400-1445 K), we follow the melting and volatile partitioning methods outlined in (20). We first compute batch melting with standard partition coefficients for CO_2 and H_2O which returns how concentrated CO_2 and H_2O are in the melt for a given melt fraction, F:

$$X_{CO_2}^{\text{melt}} = (1-(1-F))^{1/0.002}(m_{\text{mantle},CO_2}/m_{\text{mantle}})/F \quad [16]$$

$$X_{H_2O}^{\text{melt}} = (1-(1-F))^{1/0.01}(m_{\text{mantle},H_2O}/m_{\text{mantle}})/F \quad [17]$$

where m_{mantle} is the mantle mass (in kg, which for Earth is 4×10^{24} kg) and m_{mantle,CO_2} and m_{mantle,H_2O} are the masses of CO_2 and H_2O in the mantle, respectively (in kg). However, if the source material is reducing, the melt will never get this carbon rich due to graphite saturation. Therefore, we compute the carbon melt concentration assuming graphite saturation. We assume the carbon is stored in the mantle as graphite and dissolves into the melt as carbonate ions (${CO_3}^{2-}$). The amount of carbonates dissolved in the melt are calculated from equilibrium constants K_1 and K_2:

$$X_{{CO_3}^{2-}}^{\text{melt}} = \frac{K_1K_2f_{O_2}}{1+K_1K_2f_{O_2}} \quad [18]$$

$$log_{10}K_1 = 40.07639 - 2.53932\times10^{-2}T + 5.27096\times10^{-6}T^2 + 0.0267\frac{(P-1)}{T} \quad [19]$$

$$log_{10}K_2 = -6.24763 - \frac{282.56}{T} - 0.119242\frac{(P-1000)}{T} \quad [20]$$

for temperature (T, in K) and pressure (P, in bars) and f_{O_2}, which is the oxygen fugacity calculated based on the Iron-Wüstite redox buffer:

$$f_{O_2} = 10^{-27215/T+6.57+0.0552(P-1)/T} \quad [21]$$

Then, we calculate the CO_2 melt abundance:

$$X_{CO_2}^{\text{melt}} = [\frac{M_{CO_2}}{\text{fwm}}X_{{CO_3}^{2-}}^{\text{melt}}]/[1-(1-\frac{M_{CO_2}}{\text{fwm}})X_{{CO_3}^{2-}}^{\text{melt}}] \quad [22]$$

where M_{CO_2} is CO_2's molar mass and fwm is the formula weight of the melt (36.594) (20). We take the minimum of the graphite-saturated CO_2 melt concentration (Equation 22) and the constant partition coefficient concentration (Equation 16) and use that $X^{\mathrm{melt}}_{CO_2}$ value to calculate the flux of CO_2 in the melt:

$$F^{\mathrm{melt}}_{CO_2} = X^{\mathrm{melt}}_{CO_2} m_{\mathrm{melt}} F \qquad [23]$$

where m_{melt} is the planetary melt production rate (in g/s, where Earth's nominal melt production rate is 3.2×10^9 g/s). To compare the original carbon content to the amount that remains as graphite, we compute:

$$\text{Original Carbon} = \frac{m_{\mathrm{mantle},CO_2}}{m_{\mathrm{mantle}}} m_{\mathrm{melt}} \qquad [24]$$

$$\text{Remaining Graphite} = \text{Original Carbon} - F^{\mathrm{melt}}_{CO_2} \qquad [25]$$

For the Monte Carlo simulation, we vary the input parameters and sample distributions according to Table S1.

To calculate the species that would be released by magmatic outgassing and their corresponding outgassing fluxes, we use the outgassing speciation model described in (21). As magma ascends to the surface, the overburden pressure decreases and the dissolved volatiles in the magma may reach saturation. At that point, volatiles exsolve from the magma and form gas bubbles, which can be released to the atmosphere. In addition, chemical reactions take place within the bubbles which can alter their chemical compositions. This model estimates the composition of these gas bubbles just prior to their release to the atmosphere by solving a system of equations including the Iacono-Marziano solubility relationships for H_2O and CO_2, gas-phase equilibrium relationships and mass conservation of hydrogen and carbon (21). The model assumes that the oxygen fugacity of the gas is set by the oxygen fugacity of the magma and requires the following inputs: initial concentrations of H_2O and CO_2 in the melt prior to outgassing, temperature and pressure of outgassing, and redox state of the melt. Please refer to (21) for further details.

Swain et al. 2021 (22) recently claimed the detection of a thin, reducing atmosphere around the rocky exoplanet GJ 1132 b that is H_2-dominated and rich in CH_4 ($\sim$0.5%). They simulated mantle outgassing and argued that an ultra-reduced magma could reproduce the observed atmosphere, with the best fitting model parameters giving an extremely reduced oxygen fugacity of $log f_{O_2} = IW - 11$ (22). Such reduced conditions could conceivably arise if significant amounts of hydrogen from a planet's primary atmosphere dissolve into the magma ocean and were sequestered into the interior, thereby providing a reservoir of volatiles that can later be outgassed to form a secondary atmosphere (22, 23). However, the outgassing model of (22) omits carbon partitioning between solid, liquid and gas phases under ultra-reducing redox conditions.

We find that for an ultra-reduced melt of $log f_{O_2} = IW - 11$, essentially all of the carbon (>99%) will remain saturated as graphite during partial melting, so there is negligible carbon available for gaseous phases (Figure 3). To confirm this, we used the above outgassing speciation model which solves for the gas-gas and gas-melt equilibrium in a C-O-H system, to predict the gases that would be released from the melt by magmatic outgassing. From this model, we determine that the CH_4, CO_2, and CO outgassing fluxes would be negligible (<1E-10 Tmol/year) at such a reduced oxygen fugacity (Figure S2). It is important to note that these calculations do not include carbon in the form of iron carbonyls and methyl (CH_3) groups bonded to Si^{4+} in the melt as some studies have suggested will be present under reducing conditions (24, 25). However, it is not expected that these additional carbon-bearing species will significantly alter our findings as these studies also found carbon stable in the melt under reducing conditions. Therefore our findings suggest that the outgassing mechanism proposed for GJ 1132 b is improbable. Additionally, an independent study that analyzed the same Hubble Space Telescope (HST) transit data found no methane signature and instead claim a featureless spectrum for GJ 1132 b (26), and the findings of (22) conflict with Lyman-alpha observations of the system (27).

C. Calculations of Global CH_4 Flux Estimates from Abiotic Sources. The global CH_4 flux estimates for different abiotic sources in Table S2 (and illustrated in the schematic Figure 2) and Figure 4 are calculated as follows:

Higher Temperature:

- **Etiope & Lollar 2013:** To determine an estimated global abiotic CH_4 flux from surface volcanism, we take their CH_4 flux estimates for individual volcanoes and multiply these values by the number of active volcanoes on Earth today ($\sim$1500) (5).
- **Ryan et al. 2006:** To determine an estimated global abiotic CH_4 flux from surface volcanism, we followed the same procedure as described above for Etiope & Lollar 2013, except with Ryan et al.'s estimates for individual volcanic outgassing CH_4 fluxes (28).
- **Schindler & Kasting 2000:** They estimated the current global CH_4 flux from submarine volcanism to be $\sim 10^{-2}$ Tmol/year by taking an observed ratio of CH_4/CO_2 in mid-ocean ridge hydrothermal vent fluids and an estimated total outgassed carbon flux at the mid-ocean ridges.

Lower Temperature:

Observational and Theoretical Studies:

- **Cannat et al. 2010:** They derive a global serpentinization-related methane flux at slow spreading mid-ocean ridges of 2.5×10^{-2} Tmol/year (29).

- **Keir 2010:** Keir calculates a global methane flux of 2×10^{-2} Tmol/year from mid-ocean ridges, similar to the findings of Cannat et al. 2010 (30).

- **Jones et al. 2010:** Their serpentinization experiments for mid-ocean ridges and forearcs determined CH_4 production rates ranging from 1×10^{-5} to 0.05 μmol/kg/hr (31). Taking these rates and extrapolating to the mass of the entire oceanic crust, we find that the corresponding global abiotic CH_4 estimates exceed the Earth's iron supply. However McCollom 2013 (32) caution that Jones et al. did not determine their background levels of CH_4 so it is possible that a portion of the methane generated in their experiments came from sources of contamination. Therefore, the findings of Jones et al. cannot be extrapolated to a global abiotic flux.

- **Catling & Kasting 2017:** Using a combination of different observational studies, they determined abiotic CH_4 flux estimates for hot, axial vents and off-axis vent fields of 0.015 and 0.03 Tmol/year, respectively (3). For hot, axial vents, they estimated the abiotic CH_4 flux by using observed CH_4 and CO_2 concentrations from East Pacific Rise fluids. For the off-axis vent fields, they used an estimated H_2 flux and the CH_4/H_2 ratio in ultramafic vent fields to determine an abiotic CH_4 flux estimate (3).

- **Guzmán-Marmolejo et al. 2013:** They estimated the amount of CH_4 generated by serpentinization in hydrothermal vent systems. For 1$M_\oplus$ and 5 $M_\oplus$ planets, they determined abiotic CH_4 fluxes of 0.18 Tmol/year and 0.35 Tmol/year, respectively (33). These estimates account for the supply rate of available FeO in the crust which is determined in part by the crustal production rate. For the 5 $M_\oplus$ planet, the crustal production rate is scaled from Earth using a power law. They also take into account the limitations of CO_2 in hydrothermal systems based on different observational studies.

- **Kasting 2005:** Kasting 2005 estimated the global abiotic CH_4 flux from off-axis mid-ocean ridges by extrapolating from observed methane concentrations in hydrothermal fluids. They found that, at present, the abiotic hydrothermal CH_4 flux is $\sim$0.1 Tmol/year, but during the Hadean the flux may have been larger, $\sim$1.5 Tmol/year (34). They note that if seafloor creation during the Hadean was much faster, the abiotic CH_4 flux could have increased by a factor of 5-10. However, given that seafloor production rates during that period are uncertain, we only include their Hadean estimate of 1.5 Tmol/year in Figure 4 and Table S2. These estimates are about an order of magnitude larger than those of Catling & Kasting 2017 because Kasting 2005 assumed a larger water circulation rate compared to that of Catling & Kasting 2017 (3). In addition, the Kasting 2005 Hadean abiotic flux estimate is larger than Guzman-Marmolejo et al.'s estimates because Guzman-Marmolejo took into account the iron supply and CO_2 limitations in hydrothermal systems (33, 34).

- **Brovarone et al. 2017:** This study determined several different global CH_4 flux estimates for different sites where serpentinization takes place including subduction zone fluids, forearc mantle wedges above subduction zones, and sub-seafloor conditions (35).

- **Fiebig et al. 2007:** They investigated subduction-related hydrothermal sites in the Mediterranean and computed both an uppermost flux estimate for abiogenic CH_4 during the Archean (2.5-5 Tmol/year) and a present-day flux (6×10^{-3} Tmol/year) (36).

- **Fiebig et al. 2009:** This study estimated the abiogenic CH_4 flux from continental hydrothermal systems to be 0.31 Tmol/year (37).

- **Portella et al. 2019:** Their study of serpentinization of chromitites in ophiolites found that chromitites can contain CH_4 gas concentrations up to 0.31 $\mu g/g_{rock}$. Taking this CH_4 concentration and multiplying it by Earth's melt production rate (3.2×10^9 g/s) results in a global CH_4 flux estimate of 2×10^{-3} Tmol/year (38).

- **Klein et al. 2019:** They studied methane formation in olivine-hosted secondary fluid inclusions to inform serpentinization in subduction zones, mid-ocean ridges and ophiolites. They determined that the Chimaera serpentinization system has released 0.076 to 0.5 km^3 CH_4 during the past 2000 years which is equivalent to 2×10^6-11×10^6 mol/year of CH_4. They also estimated that the lower oceanic crust contains a total of $\sim$300 Tmol of CH_4 gas (39). Given that the lifetime of the oceanic crust is $\sim$200 Myrs, the estimated global abiotic CH_4 flux due to serpentinization is 1.5×10^{-6} Tmol/year.

Experiments:

- **McCollom 2013:** The hydrocarbon formation experiments discussed in McCollom 2013 measure the amount of dissolved CH_4 gas in serpentinized olivine at 300 ^oC as a function of time (see their Figure 7). Taking their experimental methane production rate of 0.05 μmol/kg/hr and scaling it to the entire mass of oceanic crust on Earth ($\sim 6 \times 10^{21}$ kg) results in a global flux estimate that exceeds Earth's iron supply (32). Such an estimate requires the whole crust to be at a high temperature which is unrealistic for a habitable zone terrestrial planet. Therefore, it is not possible to extrapolate their experimental findings to a global abiotic flux rate.

- **Oze et al. 2012:** They performed experiments to investigate the influence of mineral catalysts on serpentinization and found that the CH_4 production rate varied from $\sim$0.09 to 0.15 μmol/kg/hr. If we take their experimental CH_4 production rates and extrapolate to the mass of the oceanic crust, we find a global CH_4 flux estimate that exceeds Earth's iron supply. As with Jones et al. (31), McCollom 2013 (32) note that Oze et al. did not quantify their background levels of CH_4, so it is not possible to properly extrapolate a global abiotic CH_4 flux from their experiments.

- **Neubeck et al. 2011:** Their serpentinization experiments on forsteritic olivine determined CH_4 accumulation rates ranging from 2.7×10^{-11} to 7.3×10^{-11} mol/m^2/s. However, McCollom 2013 (32) noted that Neubeck et al. did not quantify their background CH_4 levels, so it is not possible to extract an abiotic global flux estimate from this study.

- **McCollom 2016:** They performed serpentinization experiments with olivine and measured a range of dissolved CH_4 concentrations from 5.5 to 270 μmol/kg$_{\text{olivine}}$. They used isotopic labeling to differentiate CH_4 produced by serpentinization from background sources, and found that in almost all experiments, the majority of CH_4 produced actually derived from background sources rather than from reduction of dissolved inorganic carbon. Using the isotopic labeling, for the experiments performed at or above 300 ^{o}C, the amount of CH_4 generated via reduction of inorganic carbon was 16-50 μmol/kg$_{\text{olivine}}$ (40). Taking these concentrations, dividing by the duration of the experiments and extrapolating to the mass of the oceanic crust, we find that the corresponding global abiotic CH_4 estimates exceed the Earth's iron supply. As with McCollom 2013, these experiments suggest that high temperatures are necessary to generate CH_4. Such temperatures are higher than typical temperatures for habitable zone terrestrial planets. Therefore, we cannot properly extrapolate these experimental findings to a global abiotic CH_4 flux on temperate terrestrial planets.

Impacts:

- **Kasting 2005:** Kasting estimated the global CH_4 flux due to impact events during the Hadean to be 1.24 Tmol/year (34).

- **Kress & McKay 2004:** They determined that 0.6 Tmol of CH_4 is generated by a 1-km cometary impactor (41).

- **Zahnle et al. 2020:** For a highly-reduced Pluto-sized dwarf planet impactor, they determined that it would generate ~2300 moles CH_4/cm^2 (10).

- **Court & Sephton 2009:** Experimentally studied ablation of carbonaceous chondritic materials and found that they release <100 ppm of CH_4 at temperatures up to 1000°C (42).

D. Calculations of Atmospheric Methane Lifetime for Volatile-Rich Bodies. In Figure 5, we estimate the atmospheric lifetime of methane for an Earth-mass terrestrial planet with different water mass fractions and Titan-like initial volatile inventories. Using model calculations based on Cassini data for Titan's interior composition from (43), we assume Titan's volatile content consists of 0.35 % CH_4 and ~4-6 % CO_2 relative to weight % H_2O (43). We conservatively assume that the escape flux of hydrogen is diffusion-limited and calculate the atmospheric lifetime of CH_4. First we calculate the mass of methane:

$$m_{CH_4} = (0.35/100) m_{H_2O} \quad [26]$$

where m_{H_2O} is the water mass fraction of the planet (in kg). Then we calculate the diffusion-limited escape flux of H_2.

$$\Phi = C f_T(H_2) \quad [27]$$

Φ is the escape flux of H_2 from Earth at the diffusion limit (in molecules/cm^2/s). Assuming the atmosphere is 10% CH_4, the fraction of hydrogen ($f_T(H_2)$) is 0.2 (i.e., $0.1 \times 2 = 0.2$ with two H_2 molecules per CH_4 molecule) and C for Earth's atmosphere is 2.5×10^{13} cm^{-2}s^{-1}. The atmospheric lifetime of CH_4 (in years) is given by:

$$T_{CH_4} = (\frac{m_{CH_4}}{M_{CH_4}} \text{NA})/(\Phi \times \text{SA} \times 3.154 \times 10^7) \quad [28]$$

where M_{CH_4} is the molar mass of CH_4 in mol/kg, NA is the Avogadro constant and SA is the surface area of the Earth (in cm^2). Table S3 demonstrates how the lifetime of atmospheric CH_4 increases with increasing planetary mass fraction of water. For Figure 5, we ran a Monte Carlo simulation and varied the CH_4 inventory, sampling a uniform distribution from 10^{-4} to 10^{-2} relative to weight % water, for water mass fractions from 10^{-2} to 10 weight % of the planet's mass (assuming an Earth-mass planet).

To check that our estimated CH_4 atmospheric mixing ratio of 10% is reasonable, we calculate the solubilities of CH_4 and CO_2 for the atmosphere-ocean system reservoir using Henry's Law partitioning. For CH_4:

$$(C \times m_{H_2O} \times M_{CH_4}) + m_{\text{atm}} = m_{CH_4} \quad [29]$$

where m_{H_2O} and m_{CH_4} are the masses of H_2O and CH_4, respectively. M_{CH_4} is CH_4's molar mass (kg/mol) and m_{atm} is the mass of the atmosphere, given by:

$$m_{\text{atm}} = PA/g \quad [30]$$

where P is pressure in bars, A is the surface area of the planet in m^2, and g is surface gravity (9.8 m/s^2). $[CH_4]$ is the concentration of dissolved CH_4 (mol/kg), which is given by Henry's Law:

$$[CH_4] = kP \quad [31]$$

where k is Henry's Law constant (0.0014 mol/kg/bar for CH_4). Solving for pressure, we find that for an Earth-mass planet with 1% of its mass consisting of water, the pressure of methane is ∼32 bars. Following the same formalism above for CO_2, which has a Henry's Law constant of 0.04 mol/kg/bar, its pressure is ∼22 bars. Therefore, our choice of CH_4's atmospheric mixing ratio of 10% is conservative given the volatile inventories, which also allow for plausible inventories of N_2 gas.

We also consider whether volatile-rich, habitable zone planets could produce a long-lived CH_4+CO_2 biosignature false positive if not all water is melted. The storage and slow release of CH_4 and CO_2 from clathrates (ices that trap gases) on a Titan-like planet could conceivably mimic a biosphere. If surface conditions are habitable, however, then storage of large volumes of CH_4 in pure clathrates is not possible because CH_4 clathrates are less dense than liquid water at all pressures (44). Any CH_4 clathrates stored in high pressure ices would therefore rise to the surface and rapidly dissociate. It is true that methane clathrates are a CH_4 reservoir on Earth, but this is only because they are trapped by the weight of sediments above them, and are thus in a quasi-stable state (and will be potentially perturbed by slight surface warming). The weight of sediments could not trap the $\sim 10^{22}$ mol of CH_4 required to sustain biogenic-like fluxes of CH_4 for Gyr timescales.

If surface conditions are sub-freezing, CH_4 clathrates can inhibit subsurface ocean formation at all depths, and tectonically driven ice resurfacing may continuously bring fresh clathrates to the surface, maintaining CH_4 fluxes larger than Earth's biological flux (45). The region of parameter space for which atmospheric CH_4 can be maintained is likely small, however, since clathrates are unstable against surface warming: liquid water from warming will destabilize CH_4 clathrates, causing CH4 release into the atmosphere and even more greenhouse warming (45). Initial surface temperatures must therefore be low to prevent this runaway melting. For many planets, clathrate false positives may be ruled out by estimating minimum surface temperatures from observed atmospheric gases and plausible albedos. However, additional modeling work is required to characterize clathrate-atmosphere interactions across diverse planetary conditions.

Input Parameter	Low	High	Sampling Method
Mantle Mass (M_{mantle}) (kg)	$0.1(4 \times 10^{24})$	$10(4 \times 10^{24})$	log_{10} Uniform Distribution
Mantle CO_2 Mass (M_{mantle,CO_2}) (kg)	$1 \times 10^{-5}(4 \times 10^{24})$	$1 \times 10^{-2}(4 \times 10^{24})$	log_{10} Uniform Distribution
Mantle H_2O Mass (M_{mantle,H_2O}) (kg)	$1 \times 10^{-5}(4 \times 10^{24})$	$1 \times 10^{-1}(4 \times 10^{24})$	log_{10} Uniform Distribution
Melt Fraction (F)	0.1	0.5	Uniform Distribution
Planetary Melt Production (M_{melt}) (g/s)	$0.1(3.2 \times 10^{9})$	$10(3.2 \times 10^{9})$	log_{10} Uniform Distribution

Table S1. Monte Carlo sampling distributions for carbon partitioning and gas speciation calculations.

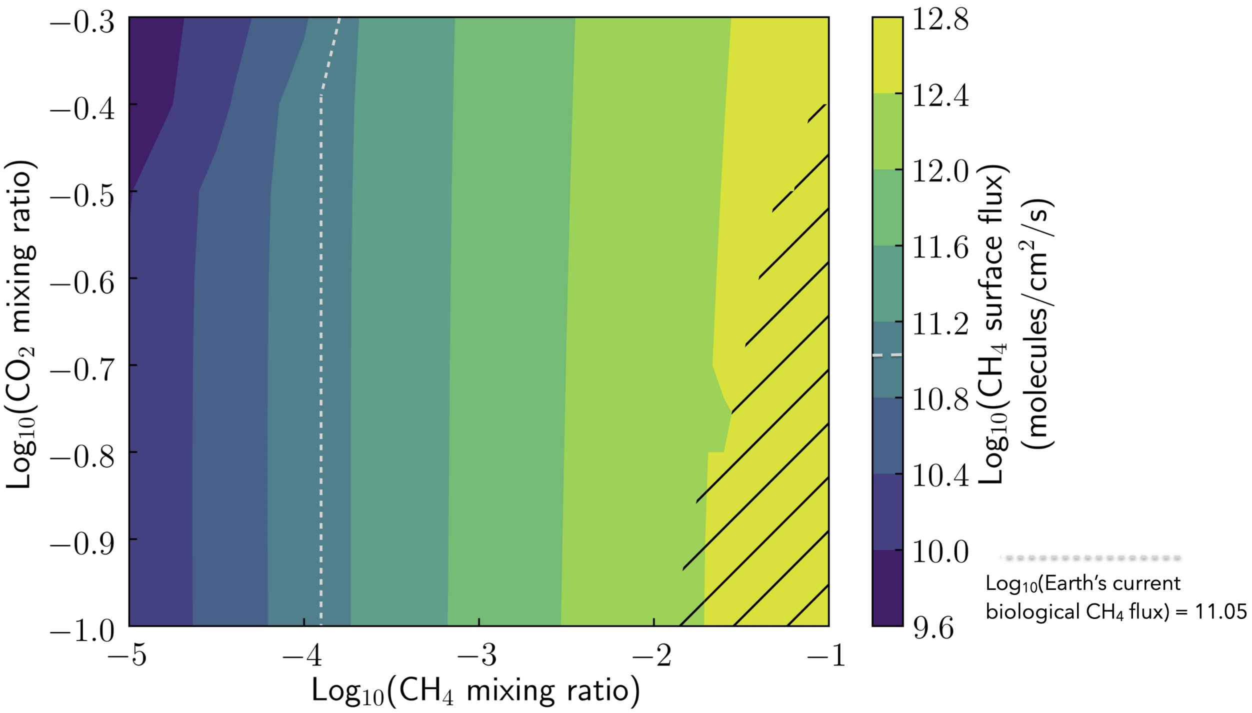

Fig. S1. Methane surface flux required to sustain CH_4- and CO_2-rich atmospheres in photochemical steady state. Using PhotochemPy, we ran a series of models with an initial atmospheric composition that is Archean Earth-like (orbiting the Sun at 2.7 Ga) exploring a range of CH_4 and CO_2 surface mixing ratios from 10^{-5} to 0.1 and 0.1 to 0.5, respectively. The contour colors correspond to the CH_4 surface flux required to sustain the atmospheric mixing ratios. While the model accounts for haze formation, we found that at higher CH_4 mixing ratios, the model had trouble converging to a steady-state solution. For those cases corresponding to the hatched region of the figure, we ran models that used the same Archean Earth-like initial atmospheric composition but removed the haze component in order to ensure model convergence. Ultimately, for abundant atmospheric CH_4 (i.e., surface mixing ratios above $\sim10^{-3}$) to be stable against photochemistry in terrestrial planet atmospheres requires a significant replenishment source that results in large CH_4 surface fluxes that are likely much larger than Earth's current biological flux.

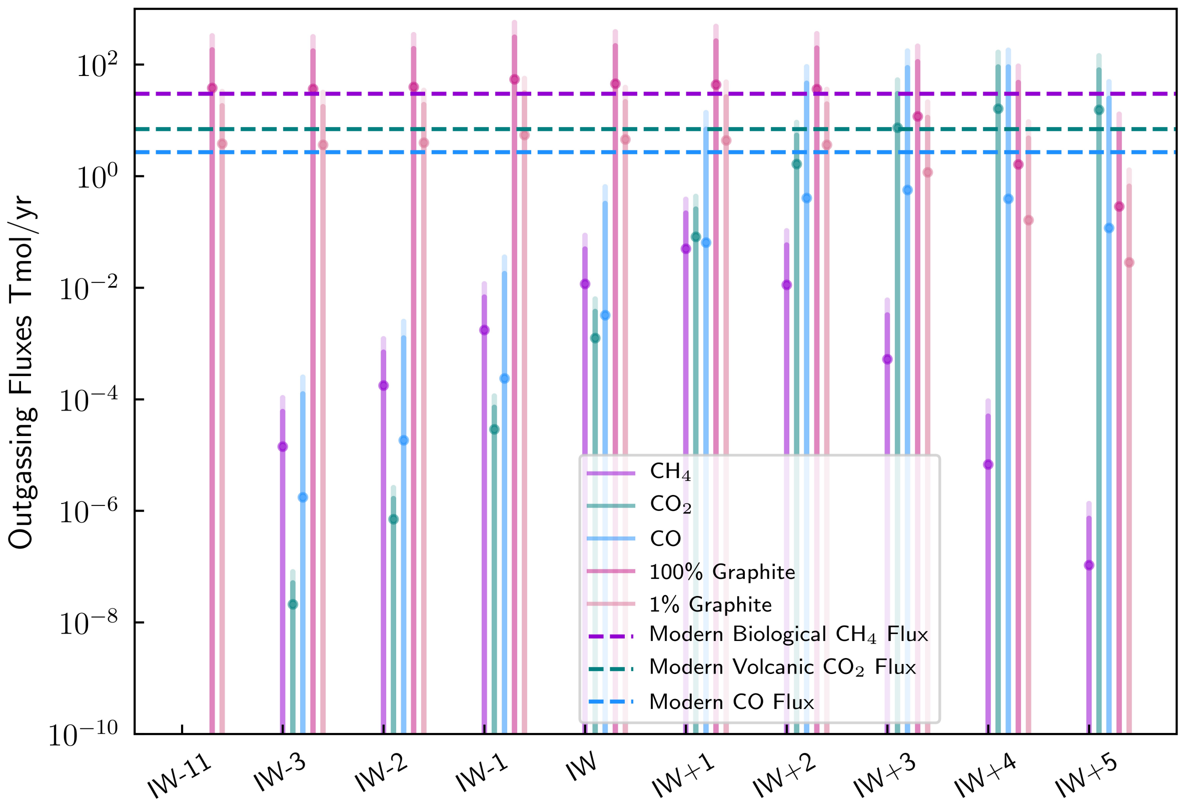

Fig. S2. Simultaneous outgassing of CH_4 and CO_2 with negligible CO is highly unlikely unless large quantities of graphite are efficiently converted to CH_4 via metamorphism. Outgassing fluxes as a function of oxygen fugacity. We used the same batch-melting model as described in Figure 3 and solved for speciation of gases produced by magmatic outgassing. The results are the average outgassing fluxes (in Tmol/year) of CH_4, CO_2 and CO from the Monte Carlo simulation with uncertainties reported as the 95% confidence intervals. The graphite results assume that either 100% or 1% of the remaining graphite can be converted into outgassed CH_4. The horizontal dashed lines show current outgassing fluxes on Earth for reference (e.g., biological CH_4 flux). For a planet with a very reduced melt composition, outgassing of any carbon species (i.e., CH_4, CO_2, and CO) will be negligible. In addition, for all oxygen fugacities considered from extremely reduced ($IW-11$) to highly oxidized ($IW+5$), the magmatic outgassing fluxes of CH_4 are still orders of magnitude lower than Earth's modern biological CH_4 flux of 30 Tmol/year.

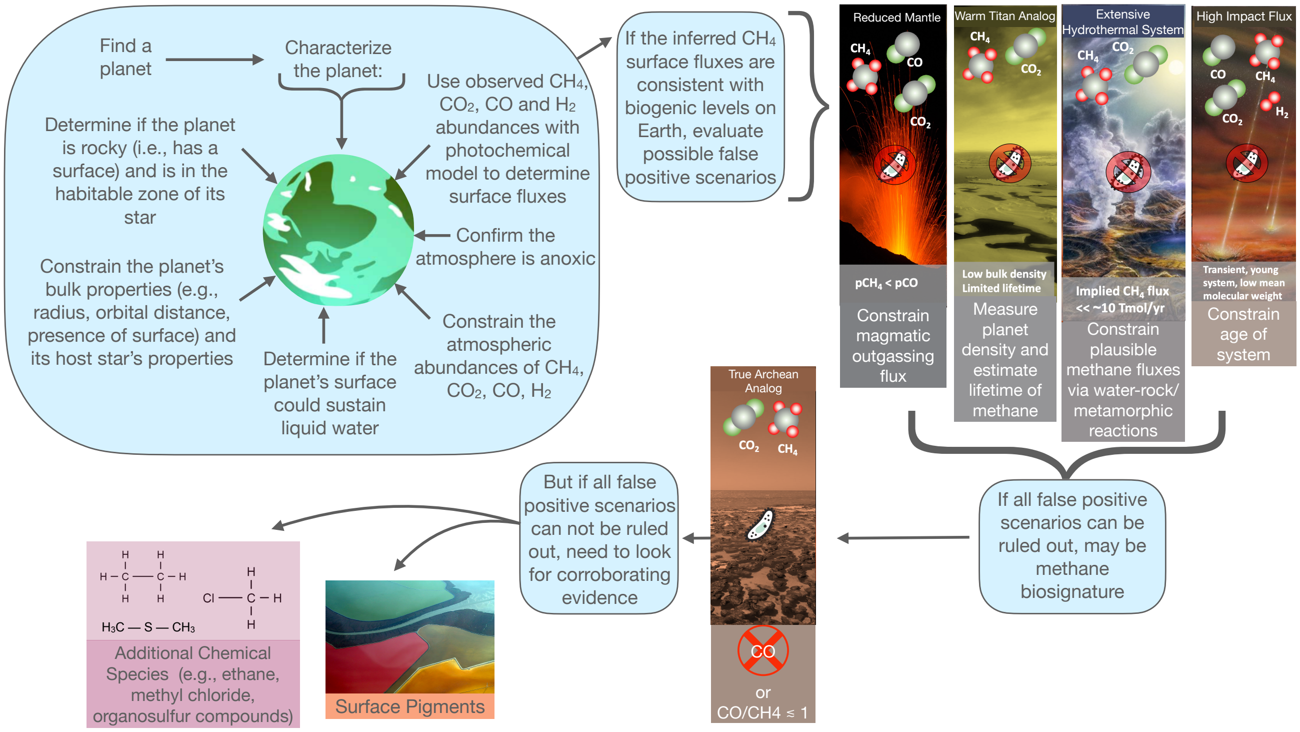

Fig. S3. Possible procedure to search for methane biosignatures on terrestrial exoplanets that takes into account the planetary context. Once an exoplanet has been detected, it is important to characterize its bulk properties (e.g., mass, radius, orbital properties, presence of a surface, host star properties). In addition, constraining its atmospheric composition, particularly the abundances CH_4, CO_2, CO, H_2, H_2O and confirming that the atmosphere is anoxic, is essential for determining the presence of a methanogenic biosphere. Using this data with a photochemical model can determine the surface fluxes of the different atmospheric constituents that are necessary to sustain the observed atmospheric abundances. If the inferred CH_4 surface fluxes are consistent with plausible biogenic levels, then all possible false positive scenarios must be evaluated. If all false positives can be definitively ruled out then a methane biosignature has been identified at a high level of confidence that must be statistically determined. However, if all false positives cannot be ruled out, then it is necessary to look for corroborating evidence like additional gas species (e.g., methyl chloride, and organosulfur compounds) and the presence of surface pigments. Credits (images): Don Dixon, Wikimedia Commons; Donald Hobern; kuhnmi; NASA/JPL-Caltech/Lizbeth B. De La Torre; Doc Searls.

Abiotic Source	Reference	CH_4 Flux	Global CH_4 Flux Estimate
**Higher Temperature**			
Volcanic	Etiope & Lollar 2013 (5)	100 tons/yr (Mt. Etna, most Icelandic volcanoes)	9.4E-3 Tmol/year
Volcanic	Ryan et al. 2006 (28)	9 tons/yr	8.4E-4 Tmol/yr
Submarine Volcanism	(46)	–	1.3E-2 Tmol/year
**Lower Temperature**			
Serpentinization at slow-spreading mid-ocean ridges	Cannat et al. 2010 (29)	3.9E7 mol/yr (Rainbow Hydrothermal Field); 0.04-0.3E7 mol/km/yr (Ridge domains with frequent ultra-mafic outcrops)	2.5E-2 Tmol/yr
Serpentinization in vent fluids from mid-ocean ridges	Keir 2010 (30)	0.1-4 mmol/kg	2E-2 Tmol/yr
Serpentinization at seafloor hydrothermal systems	Catling & Kasting 2017 (3)	–	0.015 - 0.03 Tmol/year
Serpentinization at hydrothermal vent systems	Guzmán-Marmolejo et al. 2013 (33)	–	0.18 Tmol/year (for 1 M_{Earth}), 0.35 Tmol/year (for 5 M_{Earth})
Serpentinization at off-axis mid-ocean ridges	Kasting 2005 (34)	–	0.1 Tmol/year (at present), 1.5 Tmol/year (during Hadean)
Serpentinization and carbonate reduction at subduction zones	Brovarone et al. 2017 (35)	–	9E-2 Tmol/yr (subduction zone fluids); 8E-3-0.2 Tmol/yr (forearc mantle wedges above subduction zones); 1E-2-0.1 Tmol/yr (sub-seafloor)
Serpentinization at subduction-related sites (and estimates for Archean Eon)	Fiebig et al. 2007 (36)	–	2.5-5 Tmol/yr (during Archean), 6E-3 Tmol/yr (at present)
Reduction of CO_2 in continental hydrothermal systems	Fiebig et al. 2009 (37)	–	0.31 Tmol/year
Serpentinization of chromitites in ophiolites	Portella et al. 2019 (38)	0.31 μg/g (rock) in chromitites	2E-3 Tmol/yr
Serpentinization in subduction zones, mid-ocean ridges and ophiolites	Klein et al. 2019 (39)	2E6-11E6 mol/yr (Chimaera system)	1.5E-6 Tmol/yr
Experiments of hydrocarbon formation in deep subsurface	McCollom 2013 (32)	0.05 μmol/kg/hr	CH_4 Flux > Fe supply
Serpentinization experiments for mid-ocean ridges and forearcs	Jones et al. 2010 (31)	1E-5 - 0.06 μmol/kg/hr	CH_4 Flux > Fe supply*
Serpentinization experiments investigating mineral catalysts	Oze et al. 2012 (47)	0.15 μmol/kg/hr	CH_4 Flux > Fe supply*
Serpentinization experiments on forsteritic olivine	Neubeck et al. 2011 (48)	2.7E-11-7.3E-11 mol/m^2/s	0.64-1.2 Tmol/yr*
Serpentinization experiments on olivine	McCollom 2016 (40)	$\sim$7.7E-3-1.3E-2 μmol/kg$_{olivine}$/hr	CH_4 Flux > Fe supply
**Impacts**			
Impact events during the Hadean	Kasting 2005 (34)	–	1.24 Tmol/year
Cometary impact events	Kress & McKay 2004 (41)	0.6 Tmol (generated by 1-km comet impactor)	–
Impact events for early Earth	Zahnle et al. 2020 (10)	2300 moles/cm^2 (generated by a highly-reduced Pluto-sized dwarf planet impactor)	Not applicable - transient event lasting $\sim$10,000 years
Meteorite ablation experiments	Court & Sephton 2009 (42)	$<$100 ppm from gasification of carbonaceous asteroid	–

Table S2. Summary of abiotic CH_4 sources and their estimated global CH_4 flux values. *Indicates that the experimental measurements may have over-estimated the amount of methane generated due to the presence of background sources.

Mass Fraction of Water (wt% of planet mass)	0.1	1.0	10	50
Lifetime of CH_4 (Myr)	1	10	100	500

Table S3. Estimated lifetime of atmospheric CH_4 for Earth-mass terrestrial planets with Titan-like initial volatile inventory and different size water mass fractions.

References

1. P Woitke, et al., Coexistence of ch4, co2, and h2o in exoplanet atmospheres. *Astron. Astrophys.* **646**, 10 (2021).
2. G Arney, et al., The pale orange dot: The spectrum and habitability of hazy archean earth. *Astrobiology* **16**, 873–899 (2016).
3. DC Catling, JF Kasting, *Atmospheric Evolution on Inhabited and Lifeless Worlds.* (Cambridge University Press), (2017).
4. J Krissansen-Totton, S Olson, DC Catling, Disequilibrium biosignatures over earth history and implications for detecting exoplanet life. *Sci. Adv.* **4**, 14 (2018).
5. G Etiope, BS Lollar, Abiotic methane on earth. *Rev. Geophys.* **51**, 276–299 (2013).
6. AA Giardini, CA Salotti, JF Lakner, Synthesis of graphite and hydrocarbons by reaction between calcite and hydrogen. *Science* **159**, 317–319 (1968).
7. A Reller, C Padeste, P Hug, Formation of organic carbon compounds from metal carbonates. *Nature* **329**, 527–529 (1987).
8. N Yoshida, T Hattori, E Komai, T Wada, Methane formation by metal-catalyzed hydrogenation of solid calcium carbonate. *Catal. Lett.* **58**, 119–122 (1999).
9. JR Holloway, Graphite-ch4-h2o-co2 equilibria at low-grade metamorphic conditions. *Geology* **12**, 455–458 (1984).
10. KJ Zahnle, R Lupu, DC Catling, N Wogan, Creation and evolution of impact-generated reduced atmospheres of early earth. *The Planet. Sci. J.* **1**, 11 (2020).
11. MH Wong, PR Mahaffy, SK Atreya, HB Niemann, TC Owen, Updated galileo probe mass spectrometer measurements of carbon, oxygen, nitrogen, and sulfur on jupiter. *Icarus* **171**, 153–170 (2004).
12. LN Fletcher, GS Orton, NA Teanby, PGJ Irwin, GL Bjoraker, Methane and its isotopologues on saturn from cassini/cirs observations. *Icarus* **199**, 351–367 (2009).
13. JI Moses, et al., Photochemistry and diffusion in jupiter's stratosphere: Constraints from iso observations and comparison with other giant planets. *J. Geophys. Res.* **110**, 45 (2005).
14. BJ Fulton, et al., The california-kepler survey. iii. a gap in the radius distribution of small planets. *The Astron. J.* **154**, 109 (2017).
15. X Yu, JI Moses, JJ Fortney, X Zhang, How to identify exoplanet surfaces using atmospheric trace species in hydrogen-dominated atmospheres. *The Astrophys. J.* **914**, 18 (2021).
16. JF Kasting, KJ Zahnle, JCG Walker, Photochemistry of methane in the earth's early atmosphere. *Precambrian Res.* **20**, 121–148 (1983).
17. DC Catling, JF Kasting, *Atmospheric Evolution on Inhabited and Lifeless Worlds.* (Cambridge University Press), (2017).
18. S Ranjan, et al., Photochemistry of anoxic abiotic habitable planet atmospheres: Impact of new h2o cross sections. *The Astrophys. J.* **896**, 21 (2020).
19. P Peterson, F2py: a tool for connecting fortran and python programs. *Int. J. Comput. Sci. Eng.* **4** (2009).
20. G Ortenzi, et al., Mantle redox state drives outgassing chemistry and atmospheric composition of rocky planets. *Sci. Reports* **10**, 14 (2020).
21. N Wogan, J Krissansen-Totton, DC Catling, Abundant atmospheric methane from volcanism on terrestrial planets is unlikely and strengthens the case for methane as a biosignature. *The Planet. Sci. J.* **1**, 58 (2020).
22. MR Swain, et al., Detection of an atmosphere on a rocky exoplanet. *The Astron. J.* **161**, 213 (2021).
23. ES Kite, BF Jr., L Schaefer, EB Ford, Superabundance of exoplanet sub-neptunes explained by fugacity crisis. *The Astrophys. J.* **887**, L33 (2019).
24. DT Wetzel, MJ Rutherford, SD Jacobsen, EH Hauri, AE Saal, Degassing of reduced carbon from planetary basalts. *Proc. Natl. Acad. Sci.* **110**, 8010–8013 (2013).
25. BO Mysen, K Kumamoto, GD Cody, ML Fogel, Solubility and solution mechanisms of c-o-h volatiles in silicate melt with variable redox conditions and melt composition at upper mantle temperatures and pressures. *Geochimica et Cosmochimica Acta* **75**, 6183–6199 (2011).
26. LV Mugnai, et al., Ares. v. no evidence for molecular absorption in the hst wfc3 spectrum of gj 1132 b. *The Astron. J.* **161**, 284 (2021).
27. WC Waalkes, et al., Lyα in the gj 1132 system: Stellar emission and planetary atmospheric evolution. *The Astron. J.* **158** (2019).
28. S Ryan, EJ Dlugokencky, PP Tans, ME Trudeau, Mauna loa volcano is not a methane source: Implications for mars. *Geophys. Res. Lett.* **33**, L12301 (2006).
29. M Cannat, F Fontaine, J Escartin, Serpentinization and associated hydrogen and methane fluxes at slow spreading ridges in *Diversity of Hydrothermal Systems on Slow Spreading Ocean Ridges*, eds. PA Rona, CW Devey, J Dyment, BJ Murton. (American Geophysical Union) Vol. 188, pp. 241–264 (2010).
30. RS Keir, A note on the fluxes of abiogenic methane and hydrogen from mid-ocean ridges. *Geophys. Res. Lett.* **37**, L24609 (2010).
31. CL Jones, R Rosenbauer, JI Goldsmith, C Oze, Carbonate control of h2 and ch4 production in serpentinization systems at elevated p-ts. *Geophys. Res. Lett.* **37**, L14306 (2010).
32. TM McCollom, Laboratory simulations of abiotic hydrocarbon formation in earth's deep subsurface. *Rev. Mineral. Geochem.* **75**, 467–494 (2013).
33. A Guzmán-Marmolejo, A Segura, E Escobar-Briones, Abiotic production of methane in terrestrial planets. *Astrobiology* **13**, 550–559 (2013).

34. JF Kasting, Methane and climate during the precambrian era. *Precambrian Res.* **137**, 119–129 (2005).
35. AV Brovarone, et al., Massive production of abiotic methane during subduction evidenced in metamorphosed ophicarbonates from the italian alps. *Nat. Commun.* **8**, 13 (2017).
36. J Fiebig, AB Woodland, J Spangenberg, W Oschmann, Natural evidence for rapid abiogenic hydrothermal generation of ch4. *Geochimica et Cosmochimica Acta* **71**, 3028–3039 (2007).
37. J Fiebig, AB Woodland, W D'Alessandro, W Püttmann, Excess methane in continental hydrothermal emissions is abiogenic. *Geology* **37**, 495–498 (2009).
38. Y de Melo Portella, F Zaccarini, G Etiope, First detection of methane within chromitites of an archean-paleoproterozoic greenstone belt in brazil. *Minerals* **9**, 15 (2019).
39. F Klein, NG Grozeva, JS Seewald, Abiotic methane synthesis and serpentinization in olivine-hosted fluid inclusions. *Proc. Natl. Acad. Sci.* **116**, 17666–17672 (2019).
40. TM McCollom, Abiotic methane formation during experimental serpentinization of olivine. *Proc. Natl. Acad. Sci.* **113**, 13965–13970 (2016).
41. ME Kress, CP McKay, Formation of methane in comet impacts: implications for earth, mars, and titan. *Icarus* **168**, 475–483 (2004).
42. RW Court, MA Sephton, Meteorite ablation products and their contribution to the atmospheres of terrestrial planets: An experimental study using pyrolysis-ftir. *Geochimica et Cosmochimica Acta* **73**, 3512–3521 (2009).
43. G Tobie, D Gautier, F Hersant, Titan's bulk composition constrained by cassini-huygens: Implication for internal outgassing. *The Astrophys. J.* **752**, 125 (2012).
44. G Tobie, JI Lunine, C Sotin, Episodic outgassing as the origin of atmospheric methane on titan. *Nature* **440**, 61–64 (2006).
45. A Levi, D Sasselov, M Podolak, Structure and dynamics of cold water super-earths: The case of occluded ch4 and its outgassing. *The Astrophys. J.* **792**, 125 (2014).
46. TL Schindler, JF Kasting, Synthetic spectra of simulated terrestrial atmospheres containing possible biomarker gases. *Icarus* **145**, 262–271 (2000).
47. C Oze, LC Jones, JI Goldsmith, RJ Rosenbauer, Differentiating biotic from abiotic methane genesis in hydrothermally active planetary surfaces. *Proc. Natl. Acad. Sci.* **109**, 9750–9754 (2012).
48. A Neubeck, NT Duc, D Bastviken, P Crill, NG Holm, Formation of h2 and ch4 by weathering of olivine at temperatures between 30 and 70 c. *Geochem. Transactions* **12**, 10 (2011).
